# Comparative Genomic, Metabolic and Transcriptomic Profiling of Bacterial Isolates Reveals Strain-Specific Adaptation and Safety Traits

**DOI:** 10.3390/ijms27198734

**Published:** 2026-09-30

**Authors:** Daria Barańska, Jacek Panek, Giorgia Pertile, Agata Gryta, Sylwia Różalska, Magdalena Frąc

**Affiliations:** 1 Institute of Agrophysics, Polish Academy of Sciences, 20-290 Lublin, Poland; d.baranska@ipan.lublin.pl (D.B.); j.panek@ipan.lublin.pl (J.P.); g.pertile@ipan.lublin.pl (G.P.); a.gryta@ipan.lublin.pl (A.G.); 2 Department of Industrial Microbiology and Biotechnology, Faculty of Biology and Environmental Protection, University of Lodz, 90-237 Lodz, Poland; sylwia.rozalska@biol.uni.lodz.pl

**Keywords:** microgreens, food-associated microorganisms, genomic and phenotypic traits, abiotic stress tolerance, plant growth-promoting bacteria

## Abstract

Food-associated microorganisms represent a diverse reservoir of bacterial strains with functional traits potentially relevant to host–microbiome interactions, stress resilience, metabolic processes, and microbial competition. In this study, we performed a multi-omics characterization of food-origin bacterial isolates to identify a potential candidate strain for further studies on microbiome–microgreens interactions. Over one hundred bacterial isolates were initially recovered from various food products and subjected to molecular screening, followed by whole-genome sequencing, metabolic profiling, transcriptomic characterization and morphological analysis of selected strains. Comparative characterization revealed significant differences among the strains. Among the tested isolates, strain B107/23, identified as *Priestia megaterium* (formerly *Bacillus megaterium*), showed consistently high numbers of genes associated with stress responses, nutrient uptake, plant growth-promoting traits, and colonization mechanisms. Phenotypic characterization later demonstrated high metabolic flexibility and a low substrate stress index, while transcriptomic profiling revealed the expression of genes associated with stress responses and metabolic activity. Overall, the integration of genomic, phenotypic, and transcriptomic results distinguished B107/23 as the most promising candidate among the tested isolates and revealed the molecular traits potentially relevant to interactions between the microbe and the host. These findings provide a basis for the validation of the selected strain in microgreens systems, including studies addressing plant stress resilience and post-harvest quality.

## 1. Introduction

A growing population, urbanization, and climate change are driving the search for innovative solutions in agriculture, such as urban agriculture [1]. Within this trend, microgreens, young edible plant seedlings, are attracting particular attention due to their short production cycle and exceptionally high content of bioactive compounds with proven health benefits [2,3]. Unfortunately, their delicate structure makes them extremely susceptible to abiotic stressors, particularly drought stress, which leads to rapid wilting, loss of nutritional value, and thus drastically shortens their already short shelf life [4]. Environmental microorganisms are increasingly recognized as key regulators of host-associated microbiomes, shaping community structure, influencing metabolic balance, and determining the outcome of microbial competition [5]. One promising strategy for improving plant stress resistance is the application of the holobiont concept, which views a plant as a meta-organism functioning in close integration with its associated microbiome [3,6]. This microbiome constitutes a reservoir of genes (hologenome) that can support the host in key physiological processes, including defence against abiotic stress [7].

Bacteria belonging to *Bacillus* spp. are exceptionally promising such an approach. Their widespread occurrence in diverse environmental niches, ability to form endospores, and synthesis of numerous bioactive compounds, including phytohormones, lytic enzymes, siderophores, and antimicrobial metabolites, contribute to their well-documented plant growth-promoting and stress-mitigating potential. Their ability to interact with plants and plant-associated environments involves several mechanisms, such as chemotaxis, biofilm formation, nutrient mobilization, phosphate solubilization, siderophore production, phytohormone synthesis, microbial antagonism, and many more [8,9,10,11,12,13,14,15]. Additionally, *Bacillus* spp. can affect plant responses to abiotic stresses, including drought, salinity, and oxidative stress, via synthesised phytohormones, osmoprotectants, antioxidant enzymes, and other stress-related metabolites [16,17,18]. Importantly, strains intended for research on edible plants should not only acquire desirable functional traits but should also meet safety criteria, including the absence of virulence determinants and transferable antimicrobial resistance genes. Based on this information, the initial screening performed in this study focused primarily on strains belonging to *Bacillus* spp., particularly *Bacillus subtilis* and *Priestia megaterium* (formerly *Bacillus megaterium*). However, the isolation step also yielded bacteria representing other genera, including *Klebsiella*. These isolates were preserved in the comparative analysis, because the selection of strains for potential application requires not only the identification of desirable functional traits but also the exclusion of microorganisms with potentially undesirable characteristics. Specifically, the absence of human pathogenicity-associated traits and clinically relevant antimicrobial resistance is an important criterion in terms of studies in food-related plant systems.

Despite broad research on particular bacterial strains with plant growth-promoting properties, genomic potential alone is not sufficient to predict their effect on plants. However, integrative approaches combining genomic, transcriptomic, metabolic, and phenotypic data provide significant insights into functional traits related to environmental adaptation, stress response, nutrient acquisition, colonization, microbial interactions, and safety of the strains, which are crucial for future in planta research. Such characterization can therefore be used to prioritize bacterial candidates for subsequent experimental validation in plant systems rather than as direct evidence of their effect on plant performance.

The aim of this study was to perform a comparative multi-omics screening of bacterial isolates in order to identify strains with genomic and phenotypic traits potentially relevant to future host-associated applications. Molecular identification, whole-genome sequencing, metabolic profiling, morphological characterization and baseline transcriptomic analysis were integrated to assess traits associated with environmental adaptation, nutrient acquisition, colonization-related mechanisms and antimicrobial resistance. The present study did not assess bacterial effects on plants; instead, it provides a strain-selection framework for subsequent validation in microgreen inoculation experiments.

## 2. Results

### 2.1. Molecular Identification and Phylogenetic Analysis

A total of 93 strains were subjected to PCR reactions. The identification was confirmed by the presence of bands corresponding to amplification products of 1300 bp for *B. subtilis* and 400 bp for *B. coagulans*, visible after gel visualization. Strains B56/23, B57/23, B64/23, B80/23, B99/23, B105/23 and B107/23 were preliminarily identified as *B. subtilis*. At this stage of the research, we focused only on *B. subtilis*; therefore, an analogous PCR reaction was not performed for *B. megaterium*.

All the selected 93 strains were then subjected to Sanger sequencing of the 16S rRNA gene. In most cases, the sequencing results were consistent with the preliminary PCR-based identification (Table A1). However, for several isolates, the obtained sequences showed high similarity to closely related species, including *B. velezensis*, *B. amyloliquefaciens*, *B. siamensis*, and *B. mojavensis*, making species-level identification complicated. This reflects the limited relevancy of 16S rRNA gene sequencing for closely related bacterial taxa. For isolates with a clear sequence match, a preliminary species-level assignment was reported in Table A1, whereas ambiguous results were interpreted more conservatively at the genus or species-group level. In some cases, 16S rRNA gene sequencing suggested association to the *B. subtilis* species despite a negative result received with the PCR assay. Because 16S rRNA-based identification alone may not provide sufficient resolution for closely related species, selected isolates were subjected to whole-genome sequencing to confirm their taxonomic affiliation using higher-accuracy identification. The phylogenetic analysis revealed several groups of isolates associated with the attached reference sequences (Figure 1). The *B. subtilis* reference strains were located within a cluster containing few examined isolates. *B. subtilis* NCDO 1769 was located in close proximity to B169/23, B57/23, B64/23 and B76/23, whereas *B. subtilis* SBMP4 occurred within the nearby group containing B76/23, B82/23, B56/23 and B80/23. However, bootstrap support for many internal nodes in this tree section was low, suggesting limited resolution of the 16S rRNA sequence for relationships among closely related isolates. B119/23, B98/23 and B109/23 formed a separate subgroup below the *B. subtilis*-associated cluster, while the two technical reference sequences, B110/23 and B111/23, constituted another distinct pair. Importantly, B107/23 was positioned within the group including all four attached *P. megaterium* reference strains (BPB-4, NS15, PEC-008 and 2201). Although the internal bootstrap support within this group was low, the topology was consistent with the assignment of B107/23 to *P. megaterium*. The *Staphylococcus* reference strains formed a separate lineage, whereas *Salmonella enterica* and *Klebsiella pneumoniae* clustered together with strong bootstrap support (100%).

### 2.2. Whole-Genome Sequencing

#### 2.2.1. Genome-Based Identification

Following preliminary identification by PCR and Sanger sequencing, ten isolates were selected for genome-level identification and characterization: B53/23, B54/23, B55/23, B56/23, B64/23, B80/23, B99/23, B102/23, B105/23, and B107/23. The initial screening was specifically focused on identifying *B. subtilis* isolates as potential candidates for further research; therefore, strains initially assigned to the *B. subtilis* group were prioritized for genome-level analysis. Whole-genome sequencing was used to refine their taxonomic assignment, as well as to assess genomic features relevant to their functional potential and safety. Despite strains B53/23, B54/23, and B55/23 no longer being considered potential application candidates after WGS-based identification as *K. michiganensis*, they were retained in the comparative genomic analysis to assess whether potentially beneficial functional traits co-occurred with safety-related genomic features (Table 1). This provided an important contrast for distinguishing functional potential from actual application suitability. Genomes of each strain were characterized based on the final BLAST identification, size, total gene count, number of coding sequences and number of automatically annotated genes (Table 1).

#### 2.2.2. Genome Features

The analysed genomes were screened for genes correlated with plant growth-promoting and adaptation-related functions, including nutrient acquisition, colonization, microbial interactions, pathogen defence, abiotic stress tolerance, and antimicrobial production and resistance. Comparative characterization exhibited similar functional profiles among strains assigned to the same *Bacillus* species (Figure 2), while the *K. michiganensis* strains B53/23, B54/23, and B55/23 formed a distinct group with similar functional gene patterns. In contrast to the *K. michiganensis* strains, the *B. subtilis* isolates B56/23, B64/23, B80/23, B99/23, and B105/23 revealed more balanced functional profiles. Genes associated with chemotaxis, fimbriae and pilus biosynthesis were the most abundant functional category (19–21%), followed by genes involved in nutrient acquisition (16–19%). Genes associated with stress-related and plant growth-promoting functions represented both approximately 12–14% of the analysed gene set. These strains also exhibited a lower abundance of antibiotic resistance-correlated genes (10–14%) and a higher representation of genes correlated with antimicrobial compound synthesis (4–12%) compared with the *K. michiganensis* isolates. Overall, the combination of genes associated with colonization, nutrient acquisition, stress response, and antimicrobial activity supported the selection of these *B. subtilis* strains for further comparative screening. Within the *P. megaterium* group, B107/23 revealed a functional profile characterized by a high representation of genes associated with antioxidant and lytic enzyme activity, nutrient acquisition, and chemotaxis, fimbriae and pilus biosynthesis. In the analysed *P. megaterium* genomes, these categories accounted for approximately 21–22%, 18–19%, and 19% of the considered genes, respectively. Genes related to growth-promoting processes represented approximately 11%, while genes associated with antibiotic synthesis and resistance were less abundant than in the *B. subtilis* strains. This combination of colonization, nutrient acquisition, stress response, and growth traits supported the prioritization of B107/23 for subsequent functional characterization.

#### 2.2.3. Functional Gene Profiles and Safety-Related Traits

Afterwards, the analysed genes were distributed into seven functional categories significant to plant-associated traits: Plants’ growth [19,20,21,22,23,24,25,26,27,28,29,30,31,32], Developmental processes within the plant [21,28,33,34,35,36,37,38], Plants’ tissue colonization [39,40,41,42,43,44,45,46,47,48,49,50], Interactions within the holobiont [24,37,51,52,53,54,55,56,57,58,59,60,61,62,63], Nutrient uptake [23,24,64,65,66,67,68,69,70,71], Abiotic stress resistance [19,21,22,28,30,72,73,74,75,76,77,78,79,80], and Biotic stress resistance [19,20,22,24,80,81,82,83,84,85,86,87] (Figure 3A–G). These categories represent complementary mechanisms crucial for the selection of strain for further *in planta* studies. Functional categories were designed on the basis of genome annotation followed by manual functional assessment with KEGG and literature-supported biological associations. Categories were not mutually exclusive, because individual genes may contribute to more than one ecological or physiological process. Genes assigned to Nutrient uptake may improve the accessibility of environment-located phosphorus, sulfur, iron, and nitrogen; genes involved in chemotaxis, pili, biofilm formation, and quorum sensing significantly contribute to directed movement, attachment, colonization, and persistence within the plant host. Genes related to IAA, polyamine, ethylene-related, acetoin, and 2,3-butanediol metabolism can modulate Developmental processes within the plant, whereas antioxidative enzymes, trehalose, glycine-betaine, GABA, and other stress-associated functions may contribute to Abiotic stress resistance. Lytic enzymes, siderophores, and antimicrobial compounds may support Biotic stress resistance through microbial competition and potential pathogen suppression. Across these categories, B107/23 revealed the most consistently high numbers of functionally relevant genes, ranking the highest for Plants’ tissue colonization (158 genes), Interactions within the holobiont (165), Nutrient uptake (121), and Abiotic stress resistance (132), and also showing the highest representation of genes associated with Developmental processes within the plant. Strain B55/23 exhibited the highest number of genes directly or indirectly associated with Plants’ growth (165 vs. 155 in B107/23) and the highest number of genes associated with Biotic stress resistance (116 vs. 95 in B107/23), while ranking second for Plants’ tissue colonization (155), Interactions within the holobiont (150), Nutrient uptake (115), and Abiotic stress resistance (122). B102/23 also revealed comparatively high numbers of genes correlated with Nutrient uptake and Abiotic stress resistance, whereas B53/23, B54/23, and B99/23 were grouped as strains with relatively high numbers of genes associated with Biotic stress resistance (79–82). Besides, the promising functional profiles observed in B53/23–B55/23 strains were separated from application potential because WGS identified these strains as *K. michiganensis*. In contrast, B107/23 combined a broad representation of genes across several plant-associated functional categories without the taxonomic safety concern.

Among the stress-related functional categories, B107/23 showed the highest number of genes associated with Abiotic stress resistance (132 genes), followed by B55/23 (122) and B102/23 (108) (Figure 3F). In contrast, the highest number of genes associated with Biotic stress resistance was detected in B55/23 (116), followed by B107/23 (95), while B53/23, B54/23, and B99/23 also showed comparatively high values (79–82 genes) (Figure 3G). Thus, B107/23 combined a particularly strong representation of genes potentially supporting both abiotic and biotic stress resistance. Although B53/23–B55/23 also showed high representation of stress-related functional traits, their identification as *K. michiganensis* precluded their prioritization for further application-oriented studies.

Considering the future application of the selected strains in plant and food systems, safety-related features were separated during evaluation from the beneficial functional traits. Genes associated with Antibiotic production were generally present at low levels, with the highest number detected in B99/23, followed by B64/23 and B80/23, while lower numbers were detected in B102/23, B105/23, and B107/23. The *K. michiganensis* strains B53/23–B55/23 contained few antibiotic production genes (Figure 3H). Despite antimicrobial compound production possibly being correlated with microbial competition and biocontrol, it may also create selective pressure favouring antimicrobial resistant microorganisms, which should be considered when evaluating strains for application [88,89]. Resistome profiling further exhibited that the *K. michiganensis* isolates contained a broad repertoire of determinants associated predominantly with antibiotic efflux and target alteration. These included components of several multidrug efflux and regulatory systems, including oqxA/oqxB, mdtB/mdtC, Kpn-family transporters, acr-associated determinants, marA/marR-associated determinants, and related regulatory elements (Figure 4). Single genes annotated as components of vancomycin-resistance-associated clusters, including vanY-, vanT-, vanW-, and vanG-associated determinants, were also detected. However, detecting individual cluster-associated components does not provide direct evidence of complete functional vancomycin-resistance operons. The *Bacillus*/*Priestia* strains showed a more restricted, strain-dependent resistome profile, revealing determinants of several resistance mechanisms, including antibiotic efflux, antibiotic inactivation, target alteration, target protection, and reduced permeability (Figure 4). The detected targets included, among others, ykkC/ykkD, qac-related determinants, tmrB, aadK, and individual van-associated components.

Genomic localization analysis revealed that all resistance-associated targets for which localization was assigned were chromosomal, with no targets assigned to plasmids, either in the *K. michiganensis* or *Bacillus*/*Priestia* groups (Figure 5). Notably, no AMR-associated genes or resistance-associated mutations were detected in B107/23, either on the chromosome or on plasmid-associated regions; for this reason, the strain was not included in Figure 5. No detected resistance determinants were associated with insertion sequences, prophages, or integrons in either the *K. michiganensis* or *Bacillus*/*Priestia* groups. Thus, no direct association between the identified resistome components and the analyzed mobile genetic elements was detected.

Genes associated with Pesticide/herbicide resistance were detected at low levels across all examined strains, although B107/23 contained the highest number of genes assigned to this category (Figure 3J). Overall, the safety-related genomic profile clearly distinguished B53/23–B55/23 from the remaining strains. Despite their high representation of several potentially plant-beneficial functional traits, their identification as the opportunistic pathogen *K. michiganensis*, together with their comparatively high burden of antimicrobial-resistance-associated genes, meant that these strains were not considered suitable candidates for food- or agriculture-related applications. These findings demonstrate that the presence of potentially beneficial functional traits alone is insufficient for strain prioritization and that an acceptable safety profile must constitute an essential criterion for defining a promising strain.

Overall, the comparative genomic analysis revealed that a high number of plant-associated genes alone was not sufficient to define a strain as a promising candidate. Strain prioritization was based on the combined presence of a broad repertoire of genes associated with Plants’ growth, Developmental processes within the plant, Plants’ tissue colonization, Interactions within the holobiont, Nutrient uptake, and Abiotic and Biotic stress resistance, together with metabolic pathway versatility and an acceptable genomic safety profile. B107/23 revealed the most consistently broad functional potential across these categories, including particularly high numbers of genes related to colonization, holobiont interactions, nutrient uptake, and abiotic stress resistance, while maintaining a more favourable safety-related genomic profile than the *K. michiganensis* isolates. Several *Bacillus* strains, particularly B56/23, B64/23, B80/23, B99/23, and B105/23, also combined multiple potentially beneficial functional traits and were therefore retained for further comparative evaluation. In contrast, despite the strong representation of several plant-associated functions in B53/23–B55/23, their identification as the opportunistic pathogen *K. michiganensis* and their comparatively high burden of antimicrobial-resistance-associated genes excluded them from application-oriented prioritization. Thus, the genomic screening identified B107/23 as the strongest candidate for further functional validation and demonstrated that functional potential must be considered together with safety when selecting strains for future plant inoculation studies.

### 2.3. Metabolic Profiling

Based on the molecular, genomic, and functional screening, six strains considered as the most promising candidates for further investigation were selected for metabolic phenotyping: B56/23, B64/23, B80/23, B99/23, B105/23, and B107/23.

Time-resolved AWCD profiles were created to describe the dynamics of BIOLOG reactions throughout incubation, while the area under the curve (AUC) integrated the complete time course and allowed for comparing strains quantitatively. Substrate-category kinetics in time were additionally analyzed separately for respiratory activity and biomass production, and PCA was used to investigate overall differentiation of BIOLOG profiles. Time-resolved AWCD profiles revealed strain-dependent differences throughout incubation (Figure 6). For metabolic activity at 590 nm, most strains exhibited a rapid increase in time period between 10 and 25 h, followed by a relatively stable phase, while B107/23 strain showed a slower increase and consistently lower AWCD values (Figure 6A). The 750 nm profiles were more variable and showed less pronounced differentiation among strains (Figure 6B). A similar distinction was observed for chemical sensitivity: B107/23 remained at comparatively low AWCD values throughout incubation, particularly at 590 nm, whereas the remaining strains generally showed stronger increases over time (Figure 6C,D). AUC analysis confirmed that the differences between strains were strongly dependent on substrate category and wavelength (Figure 7). At 590 nm, B107/23 showed the lowest cumulative responses for amino acids and carboxylic acids and was significantly lower than the remaining strains in both categories. It also revealed lower AUC values for amines and amides than B105/23 and B99/23 and the lowest AUC for carbohydrates, while B80/23 showed the highest value for this substrate category. No statistically significant differences among strains were detected for polymers or the “other” substrate category (Figure 7A). At 750 nm, B107/23 and B99/23 exhibited the lowest AUC values for amino acids, while B107/23 and B99/23 created the lower statistical group for amines and amides compared with B105/23 and B56/23. Differences in the remaining substrate categories showed more complex, partially overlapping statistical groupings (Figure 7B).

Substrate-category kinetics over time further demonstrated differences in substrate utilization. For respiratory activity, polymers showed the highest relative utilization during the early phase of incubation, with the strongest response observed for B107/23. This was followed by increased utilization of amines and amides, with the most pronounced rise and decline observed for B99/23. Amino acid utilization increased later and remained relatively sustained, whereas B107/23 showed the lowest utilization of this substrate group. Carbohydrates and carboxylic acids were utilized at moderate and stable levels throughout incubation (Figure A1A,C,E,G,I,K). Biomass-associated substrate utilization revealed less pronounced variation. Polymers contributed to metabolic activity during the early phase, again with the highest utilization observed for B107/23, while carbohydrate utilization remained stable. Amino acids and carboxylic acids contributed less to biomass production, with particularly low values observed for B99/23 and B107/23 (Figure A1B,D,F,H,J,L). These profiles indicate that strain differentiation involved not only the quantity of substrate utilization but also the timing, with the strongest differences associated with polymers, amines and amides, amino acids and carboxylic acids. PCA confirmed strain-dependent differences in the BIOLOG profiles (Figure A2). For carbon-source utilization at 590 nm, PC1 and PC2 explained 76.5% of the total variance, with B80/23 and B107/23 clearly separated from the remaining strains (Figure A2A). At 750 nm, the first two components explained 63.0% of the variance and revealed a broader dispersion of strain profiles, indicating greater differentiation in biomass-associated responses (Figure A2B). A similar separation was observed for chemical sensitivity at 590 nm, where PC1 and PC2 explained 65.1% of the variance and B80/23 and B107/23 occupied positions distinct from the more closely grouped B56/23, B64/23, B99/23, and B105/23 (Figure A2C). For chemical sensitivity at 750 nm, the first two components accounted for 51.3% of the variance; B107/23 remained separated from most strains, while B64/23 showed a comparatively broad within-strain dispersion (Figure A2D). Overall, the PCA demonstrated clear strain-specific variation in both carbon-source utilization and responses to inhibitory compounds, with B107/23 showing one of the most consistently distinct phenotypic profiles across the analysed BIOLOG parameters.

Carbon-source utilization profiles were evaluated after 72 h of incubation using the individual carbon compounds present in the GEN III MicroPlate wells as metabolic substrates. At 590 nm, reflecting respiratory activity associated with substrate utilization, the strains revealed various metabolic profiles (Figure 8A). B80/23 strain exhibited high respiratory responses to numerous carbon sources, such as gelatin, D-salicin, L-alanine, dextrin, D-galacturonic acid, maltose, gentiobiose, trehalose, and glucose. B56/23, B99/23, and B105/23 generally exhibited moderate respiratory activity, with stronger reaction to particular substrates, while B64/23 displayed low-to-moderate activity, with a pronounced response to lactic acid. B107/23 exhibited the most even respiratory profile, with relatively low-to-moderate responses across the tested carbon sources. Measurements at 750 nm, reflecting biomass-associated turbidity, exposed a different pattern (Figure 8B). B64/23, B80/23, and B107/23 exhibited increased biomass production on particular carbon sources, while B105/23 showed a strong response to inosine. B99/23 exhibited the lowest overall biomass-associated signal. The differences between the 590 and 750 nm profiles indicate that a strong respiratory response to a particular carbon source did not necessarily result in proportionally high biomass production, which reflects variations in the balance between substrate oxidation and biomass formation. To integrate these two responses, the Substrate Stress Index (SST; A590/A750) was calculated to express respiratory activity relative to biomass production. Higher SST values indicate greater substrate-associated respiratory activity in relation to biomass formation, whereas lower SST values indicate proportionally greater conversion toward biomass [90]. Overall, SST was interpreted as an indicator of the balance between respiratory activity and biomass production, rather than as a direct measure of stress tolerance or environmental adaptability. Across the tested carbon sources, B56/23, B80/23, and B99/23 generally showed higher SST values. In contrast, B64/23 and B107/23 exhibited low-to-moderate SST values, with higher responses restricted to selected substrates. B64/23 showed the lowest overall SST profile among the examined strains (Figure 8C); however, strain B107/23 also exhibited a very low and balanced overall SST profile.

Chemical sensitivity profiles revealed strain-specific responses to the inhibitory compounds present in the GEN III MicroPlates (Figure 9). At 590 nm, most strains exhibited low-to-moderate respiratory activity after 72 h of incubation forced by the presence of chemical stressors, which indicates the inhibition of respiratory metabolism under the tested compounds. B64/23 displayed an intermediate response to several compounds, whereas B56/23, B80/23, B99/23, B105/23, and B107/23 generally showed reduced respiratory activity in the presence of antibiotics and redox-active compounds (Figure 9A). Biomass-associated responses at 750 nm were similarly low under most inhibitory conditions, indicating limited biomass production in the presence of these stressors (Figure 9B). The SST further differentiated the strains by describing the balance between respiratory activity and biomass formation under chemical stress. Lower SST values in B107/23 indicated a comparatively more balanced relationship between these processes, whereas higher values observed in other strains reflected proportionally greater respiratory activity relative to biomass production.

Overall, the BIOLOG analyses showed that the examined strains differed in the intensity and timing of their metabolic responses. The observed differences were substrate specific and did not reflect a simple ranking of overall metabolic activity. B107/23 was distinguished by a characteristic temporal profile, including pronounced early polymer utilization, comparatively low cumulative responses to selected substrate groups, and a distinct position in the multivariate analyses. These patterns were consistent across the kinetic profiles, AUC-based summaries, and PCA. In parallel, the respiration-to-biomass profiles showed that respiratory activity was not consistently proportional to biomass formation. B107/23 and B64/23 displayed the most balanced metabolic profiles, whereas several other strains exhibited high respiration-to-biomass ratios indicative of less efficient conversion of substrate utilization into biomass. Thus, the BIOLOG analysis identified distinct strain-specific strategies of substrate utilization and resource allocation, with B107/23 representing a metabolically distinct and comparatively stable phenotype rather than a universally more active one.

### 2.4. Transcriptome Sequencing of Selected Strains

Two strains, B56/23 and B107/23, were selected for transcriptomic analysis because they represented the most relevant application candidates emerging from the preceding genomic and phenotypic screening. Initial comparison of the 80 most highly expressed genes indicated that both strains shared strong expression of core functions related to translation, DNA maintenance, cell-envelope processes, transport, central metabolism, and stress responses, but differed in the relative contribution of these functional groups. B56/23 showed a stronger representation of stress-, repair-, and sporulation-associated functions, whereas B107/23 showed a broader representation of metabolic, structural, redox, and cellular-maintenance functions. Differential expression analysis of orthologous groups revealed clear transcriptomic differentiation between B56/23 and B107/23, with numerous significantly differentially expressed groups detected in both directions (Figure 10A). Functional comparison at the KEGG pathway level further showed marked differences between the strains (Figure 10B). Most of the top 20 pathways showing the largest expression differences were shifted towards B107/23, including Ribosome, Glycolysis/Gluconeogenesis, Pyruvate metabolism, Peptidoglycan biosynthesis, Vitamin B6 metabolism, and pathways related to amino acid metabolism and biosynthesis, including Phenylalanine, tyrosine and tryptophan biosynthesis and Cysteine and methionine metabolism. In contrast, non-homologous end-joining showed higher expression in B56/23, indicating a stronger contribution of this DNA repair-associated pathway. These pathway-level differences were broadly consistent with the preliminary screening of the most highly expressed genes. B56/23 showed a stronger representation of selected repair-, protein-quality-control-, and sporulation-related functions in the top-gene analysis, whereas B107/23 showed a broader representation of metabolic, redox, structural, and cellular-maintenance functions. Overall, the analyses confirmed clear functional differentiation between the two strains, with B107/23 showing a broader representation of highly expressed metabolic and biosynthetic pathways among the top differentially represented KEGG categories.

### 2.5. Morphological Characterization

Holotomography imaging of the tested strains revealed that B107/23 formed markedly larger rod-shaped cells than the other examined strains, frequently arranged in short chains and multilayered structures, with heterogeneous intracellular density (Figure 11). Descriptive morphological characteristics and approximate cell dimensions of the examined strains are presented in Figure 11, while additional morphological characteristics are summarized in Table A3. These features distinguished B107/23 morphologically from the remaining isolates and were consistent with its taxonomic assignment. Overall, holotomography confirmed the distinct cellular morphology of B107/23 and provided an additional phenotypic characteristic supporting its differentiation from the other selected strains.

## 3. Discussion

The integrated analysis revealed three principal findings. First, preliminary PCR and 16S rRNA-based identification was insufficient for reliable species-level assignment, as WGS reassigned several isolates, including B53/23–B55/23, to *K. michiganensis*. Second, the analyzed taxonomic groups differed markedly in their functional and safety-related genomic profiles. *B. subtilis* isolates showed relatively conserved colonization-, nutrient acquisition-, and stress-associated traits, whereas *P. megaterium* B107/23 combined a broad repertoire of potentially adaptive functions with a balanced metabolic phenotype. In contrast, the *K. michiganensis* isolates contained a comparatively high abundance of antimicrobial resistance determinants and should not be considered candidates for food-associated applications. Third, integration of genomic, metabolic and baseline transcriptomic data prioritized B107/23 for subsequent experimental validation. However, because plant inoculation experiments were not performed, the present results demonstrate candidate potential rather than plant-growth-promoting activity. The discrepancy between marker-based identification and WGS is particularly relevant for strain screening intended for food-associated applications. Closely related or incorrectly assigned isolates may appear acceptable at the preliminary screening stage, whereas genome-level identification can reveal taxonomic and safety characteristics that fundamentally alter their suitability for further development. Thus, 16S rRNA sequencing should be considered a preliminary screening tool in this workflow rather than a definitive basis for species-level identification or candidate selection. The limitations observed in the present study are consistent with previous reports showing that, although 16S rRNA gene sequencing is a valuable tool for bacterial identification, its discriminatory power may be insufficient for reliable species-level assignment in some closely related bacterial taxa [91,92].

WGS revealed that the isolates represented functionally distinct taxonomic groups rather than a homogeneous collection of *Bacillus* strains. The *B. subtilis* isolates showed the most conserved genomic profiles, characterized by relatively similar representation of chemotaxis-, adhesion-, nutrient acquisition-, and stress-associated genes. This is consistent with the ecological versatility commonly attributed to *B. subtilis*. In contrast, *P. megaterium* isolates, particularly B107/23, showed greater representation of functions related to antioxidant defence, extracellular enzymatic activity and nutrient acquisition. The *K. michiganensis* isolates formed a clearly different group, characterized by a comparatively high proportion of antimicrobial resistance determinants. Therefore, the value of including these isolates in the comparative analysis lies primarily in demonstrating the importance of genome-level identification and safety screening rather than in identifying them as candidates for application. The *B. subtilis* isolates exhibited comparatively conserved functional profiles, supporting the concept that core mechanisms of environmental sensing, motility, nutrient acquisition and stress adaptation are broadly maintained within this species. Nevertheless, strain-level differences were apparent in metabolic phenotyping and baseline transcriptional activity, indicating that taxonomic identity alone is insufficient for selecting an optimal candidate [93]. Thus, although the *B. subtilis* isolates possessed several traits previously associated with plant-associated lifestyles, their relative suitability should be assessed at the strain rather than species level [94]. This interpretation is consistent with the broad metabolic and environmental-response capacity described for *B. subtilis*, including the utilization of diverse carbon sources, transport systems, motility and chemotaxis [95,96]. The potential relevance of chemotaxis-related traits to plant association is further supported by experimental evidence demonstrating that intact chemotaxis machinery contributes to the early colonization of *Arabidopsis thaliana* roots by *B. subtilis* [97]. Previous genome analyses have demonstrated the substantial genomic and metabolic capacity of *P. megaterium* [98], while studies of plant-associated strains have identified genes associated with stress adaptation, nutrient acquisition, rhizosphere competence and other plant growth-promoting functions [99,100,101]. Importantly, these similarities at the genomic level do not demonstrate that B107/23 produces the same plant-beneficial effects. Rather, they show that its genomic repertoire contains functional categories previously identified in experimentally characterized *P. megaterium* strains and therefore provide a rationale for its subsequent plant-based validation.

Safety screening substantially changed the interpretation of the comparative dataset. Although B53/23–B55/23 contained several genes that could superficially be categorized as nutrient acquisition-, stress-, or interaction-related, WGS identified these isolates as *K. michiganensis*, and their genomes contained a comparatively high abundance of antimicrobial resistance determinants. Therefore, functional potential cannot be considered independently of taxonomic and safety information. In the context of edible crops, the presence of potentially desirable metabolic traits does not outweigh safety concerns associated with opportunistic pathogenic taxa. Antimicrobial resistance determinants occurring in plant-associated environments may contribute to the environmental resistome and represent an additional criterion that should be considered during candidate selection [89]. Moreover, the relevance of species-level identification is reinforced by reports of food-associated *K. michiganensis* isolates carrying clinically relevant antimicrobial resistance determinants, including carbapenemase genes [102]. Thus, the recovery of *K. michiganensis* isolates in the present screening highlights the value of integrating taxonomic identification with genome-based safety assessment at an early stage of candidate selection. The final interpretation of the resistance profiles of the analyzed isolates should additionally consider the identity of the detected antimicrobial resistance determinants and their genomic context, particularly whether they are associated with potentially mobile genetic elements. These aspects are relevant to distinguishing intrinsic resistance-related features from determinants that may have greater potential for horizontal dissemination. In the present study, the detected resistance-associated targets were predominantly chromosomal, with no determinants assigned to plasmids or associated with insertion sequences, prophages, or integrons. This genomic context suggests that the observed resistance profiles were mainly associated with chromosomally encoded features rather than with readily identifiable mobile resistance elements, which is relevant because linkage to mobile genetic elements may increase the potential for horizontal dissemination of antimicrobial resistance determinants [89,103]. However, the absence of detectable association with mobile elements does not eliminate the safety concerns related to the *K. michiganensis* isolates, which carried a substantially broader repertoire of resistance-associated determinants, including multiple multidrug-efflux and regulatory systems. In contrast, the more restricted resistome observed in the *Bacillus*/*Priestia* group, and particularly the absence of detectable AMR-associated genes or resistance-associated mutations in B107/23, provides an additional safety-related argument supporting its prioritization for further validation. Thus, the present results indicate that resistance profiles should be evaluated not only on the basis of the total number of detected determinants, but also according to their functional identity, genomic localization, and potential association with mobile genetic elements.

Importantly, B107/23 was not prioritized on the basis of a single genomic trait, but because independent genomic, metabolic and transcriptional datasets converged on a phenotype characterized by broad metabolic capacity and comparatively balanced stress-associated regulation. At the genomic level, B107/23 showed a broad representation of functions potentially related to nutrient acquisition, extracellular enzymatic activity and environmental adaptation. Previous genomic analyses have demonstrated the extensive metabolic capacity of *P. megaterium* [98], while experimental multi-omics studies have shown that this species can reorganize central metabolic pathways in response to imposed environmental stress [104]. These literature data provide biological context for the functional categories identified in B107/23, but do not demonstrate that the same mechanisms are active under the conditions examined in the present study. Metabolic phenotyping provided an additional level of strain differentiation. Importantly, the BIOLOG results did not indicate a simple ranking of strains according to overall metabolic activity, but rather revealed strain-specific differences in substrate utilization [105,106] and in the relationship between respiratory activity and biomass formation. The relatively low respiration-to-biomass ratio observed for B107/23 suggests a more balanced relationship between respiratory activity and biomass accumulation under the tested conditions. This parameter should not be interpreted as a direct measure of environmental stress tolerance; rather, it provides an operational indication of how efficiently respiratory activity is coupled to growth across the tested substrates. The kinetic analysis further demonstrated that differences among the strains involved not only the magnitude of the metabolic response but also its temporal organization, supporting the presence of strain-specific substrate utilization strategies. Together with its distinct kinetic and multivariate profile, this suggests that B107/23 differs from the remaining strains primarily in the organization of its metabolic response rather than in universally higher substrate utilization. Such a phenotype may reflect strain-specific allocation of carbon among alternative metabolic routes, as substrate-dependent partitioning of carbon flux and rearrangement of central metabolism have previously been demonstrated in B. megaterium [104,107]. Thus, the BIOLOG data complement the genomic characterization by demonstrating a distinct metabolic phenotype, while not allowing direct inference of plant-associated performance or stress tolerance. Under the standardized cultivation conditions used for RNA sequencing, B107/23 showed relatively high expression of genes associated with redox homeostasis, membrane maintenance, central metabolism and regulatory processes. The subsequent differential expression analysis of orthologous groups and pathway-level comparison further demonstrated functional differentiation between B107/23 and B56/23. At the functional level, B107/23 showed a broader baseline transcriptional representation of metabolic, biosynthetic and cellular-maintenance processes, whereas B56/23 showed a comparatively stronger contribution of selected repair-associated functions. These patterns indicate constitutive or baseline investment in functions potentially relevant to environmental adaptation; however, they do not demonstrate an induced stress response because no stress-versus-control transcriptional comparison was performed. Previous transcriptomic studies have shown that stress-associated expression in *Bacillus* is strongly dependent on the imposed environmental condition [104,108,109]. Accordingly, the transcriptional profile observed here should be regarded as baseline functional characterization rather than evidence of superior stress tolerance. Morphological differences observed by holotomography were broadly consistent with taxonomic differences among the isolates. However, morphology alone does not allow inference of colonization efficiency or stress tolerance, and these observations should therefore be regarded as complementary phenotypic characterization.

Taken together, the study demonstrates the value of integrating genome-level identification, functional annotation, metabolic phenotyping and transcriptomic profiling as a pre-screening strategy for bacterial candidate selection. Among the analyzed isolates, B107/23 showed the most consistent combination of genomic and phenotypic traits potentially relevant to environmental adaptation and host-associated interactions. This finding justifies prioritizing B107/23, together with selected B. subtilis comparators, for subsequent microgreen experiments. Such experiments should directly assess colonization efficiency, plant growth and physiological responses, tolerance to defined abiotic stresses, effects on the endogenous microbiome, post-harvest quality, and strain-level safety. Until these effects are experimentally demonstrated, B107/23 should be regarded as a candidate strain rather than a validated plant-beneficial inoculant. Genomic and transcriptomic analyses can prioritize strains and generate mechanistic hypotheses, but cannot replace direct plant inoculation experiments.

## 4. Materials and Methods

### 4.1. Isolation and Screening of Bacterial Strains

Bacterial isolates used in this study were obtained from dairy and pickled food products. The origin of investigated isolates is detailed in Table A1. Over 100 strains were initially isolated from various dairy and pickled products. Since the isolates were obtained from food matrices rather than soil or field environments, soil and climatic conditions were not applicable to their collection. The dairy sources included cow’s powdered milk (Krasnystaw, Siedlce, Mlekovita, SM Gostyń, Poland); goat’s powdered milk (Danmis, Bukowiec, Poland); powdered unmineralized acid whey produced during cottage cheese production (Krasnystaw, Poland); microfiltered fresh pasteurized milk (3.2%) (OSM Garwolin, Wola Rębkowska, Poland); unsweetened condensed UHT milk (7.5%) (Łaciate, “MLEKPOL”, Grajewo, Poland); and milk retentate (Krasnystaw, Poland). Pickled sources included homemade pickled and fresh-pickled cucumbers, pickled radish (Zakiszony, Warsaw, Poland and Sznajder, Karnice, Poland), pickled radish with thyme (Sątyrz, Poland), homemade cucumber ferment, homemade pickled beets, and commercially pickled beets (Runoland, Góra, Poland and Lokalny Warzywniak, Warsaw, Poland). Each dairy sample was suspended in 0.85% saline solution and serially diluted to obtain 10^−1^, 10^−2^, 10^−3^, 10^−4^, and 10^−5^ dilutions using sterile Milli-Q water. Pickled products were sliced, homogenized, suspended in 0.85% saline, and diluted similarly. Each dilution was then cultured on *Bacillus* cultivation medium (BC) and Plate Count Agar (PCA) (Biomaxima, Lublin, Poland), using 100 µL of inoculum, and incubated at 23 °C. The isolation strategy was designed as an initial broad cultivation and screening step rather than as a strictly selective isolation procedure for *Bacillus* spp. Although the primary interest was focused on bacteria morphologically resembling *Bacillus* spp., the use of BC medium together with the non-selective PCA medium allowed recovery of a broader range of cultivable bacteria present in the investigated food matrices. Candidate colonies were subsequently pre-selected based on colony morphology and subjected to molecular identification. Consequently, isolates belonging to genera other than *Bacillus*, including *Klebsiella*, were also recovered and retained for comparative molecular and genomic characterization. Their inclusion enabled both the assessment of functional diversity among the recovered isolates and the exclusion of unsuitable candidates during subsequent safety-oriented screening. The BC medium composition per liter was as follows: 5 g of yeast extract (Biocorp, Warsaw, Poland), 5 g of bacterial peptone (Biocorp, Warsaw, Poland), 5 g of D-glucose (Sigma-Aldrich, St. Louis, MO, USA), 0.5 g K_2_HPO_4_ (Chempur, Poland), 0.3 g KH_2_PO_4_ (Chempur, Poland), 0.3 g MgSO_4_ (Chempur, Piekary Śląskie, Poland), and 1 mL of microelement solution with the following composition: 10 mg mL^−1^ NaCl, 18 mg mL^−1^ FeSO_4_ × 7H_2_O, 16 mg mL^−1^ MnSO_4_ × H_2_O, 1.6 mg mL^−1^ ZnSO_4_ × 7H_2_O, 1.6 mg mL^−1^ CuSO_4_ × 5H_2_O, and 1.6 mg mL^−1^ CoSO_4_ × 7H_2_O in 1 L of water. After 24 h of incubation, colonies were selected based on their morphology. Colonies morphologically resembling typical *B. subtilis* (generally round, irregular, or wrinkled, with a dry or rough surface; off-white, cream, or light yellow in colour; and flat or slightly raised elevation) were pre-identified visually. Selected colonies underwent three rounds of sub-culturing to obtain pure cultures. Most of the selected strains displayed robust growth on the BC medium, which was then used as the cultivation medium in further experiments. A total of 93 bacterial strains were selected (B47/23–B65/23, B75/23–B85/23, B86/23–B99/23, B100/23–B109/23, B112/23–B125/23, B127/23, B133/23, B135/23, B137/23, B139/23, B141/23, B147/23, B149/23, B150/23, B152/23–B153/23, B160/23–B161/23, B168/23–B171/23, B175/23–B176/23, B180/23, B183/23, B185/23–B186/23, B189/23, B192/23).

### 4.2. DNA Extraction and PCR Amplification of Isolates

Bacterial biomass for DNA extraction was obtained by harvesting colonies grown on agar plates following 24 h of incubation at 23 °C. Genomic DNA was extracted from all 93 bacterial isolates using PrepMan Ultra Sample Preparation Reagent (Applied Biosystems, Foster City, CA, USA), according to the manufacturer’s protocol. The concentration and purity of the isolated DNA were verified using a NanoDrop-2000 Spectrophotometer (Thermo Scientific, Wilmington, DE, USA).

Two PCR amplifications were performed separately, targeting two molecular markers: the endoglucanase gene [110] and the *comK* gene [111], using the Veriti Fast thermal cycler (Applied Biosystems, Foster City, CA, USA). The endoglucanase gene was selected to pre-identify *B. subtilis* from the total number of isolates. The touchdown PCR reaction was prepared in a 10 µL mixture per sample, consisting of REDTaq PCR Mix (Sigma-Aldrich, St. Louis, MO, USA), deionized water, EN1F (5′-CCAGTAGCCAAGAATGGCCAGC-3′) and EN1R (5′-GGAATAATCGCCGCTTTGTGC-3′)’ primers [110], and the DNA (diluted to 2 ng µL^−1^). The protocol was as follows: The initial denaturation step at 94 °C for 3 min, followed by 10 amplification cycles: 94 °C for 15 s, 70 °C for 20 s (each cycle, a 1 °C decrease in the annealing temperature), and 74 °C for 45 s. Subsequently, other 25 cycles were performed at 94 °C for 15 s, 60 °C for 30 s, and 74 °C for 45 s, with a final extension at 74 °C for 10 min.

The use of the *comK* gene as a PCR marker enabled the pre-identification of *Bacillus coagulans* among the total number of isolates. The reaction was conducted in a 10 µL mixture per sample, containing REDTaq PCR Mix (Sigma-Aldrich, St. Louis, MO, USA), deionized water, BCR (5′-GAAACGATGGCGCTGGATAC-3′) and BCF (5′-GATCATTTTCTTCCATGGTATG-3′) primers [111], and the DNA (diluted to 2 ng µL^−1^) on the Veriti Fast thermal cycler (Applied Biosystems, Foster City, CA, USA). The protocol included: the initial denaturation step at 94 °C for 3 min, followed by 45 amplification cycles: 94 °C for 15 s, 60 °C for 30 s, and 72 °C for 45 s, and ending with a 10 min extension at 74 °C.

The V6–V9 region of the 16S rRNA gene was amplified as a preliminary molecular identification step and to generate PCR products suitable for subsequent Sanger sequencing. Amplification was performed in a 10 µL reaction mixture containing 5 µL of REDTaq PCR ReadyMix (Sigma-Aldrich, St. Louis, MO, USA), 2.8 µL of deionized water, 0.1 µL of each primer, 939F and 1492R [112], and 2 µL of template DNA diluted 1:100. The final primer concentration was 0.2 µM. PCR was carried out using a Veriti Fast Thermal Cycler (Applied Biosystems, Foster City, CA, USA) under the following conditions: initial denaturation at 94 °C for 3 min; 35 cycles of 94 °C for 15 s, 55 °C for 30 s, and 72 °C for 45 s, followed by a final extension at 72 °C for 7 min. The resulting amplicons were used in the next step for Sanger sequencing-based taxonomic identification of the isolates.

Electrophoresis was performed using a horizontal electrophoresis system and PowerPac HC High-Current Electrophoresis Power Supply (Bio-Rad, Hercules, CA, USA), and DNA bands were visualized under ultraviolet (UV) light using a Molecular Imager^®^ Gel Doc™ XR+ System (Bio-Rad, Hercules, CA, USA), with SimplySafe (EURx, Warsaw, Poland) as the staining agent.

### 4.3. Sanger Sequencing and Phylogenetic Analysis

Sanger sequencing was performed to confirm the preliminary identification of the strains by PCR. To conduct the sequencing, the BigDye™ Terminator V1.1 Cycle Sequencing Kit was used (Applied Biosystems, Foster City, CA, USA) with the following primers: 939F and 1492R [113], amplifying the V6–V9 hypervariable regions of the bacterial 16S rRNA gene. Sequencing was performed using the Applied Biosystems 3130 Genetic Analyzer (Foster City, CA, USA). Raw sequencing data were assessed for quality, trimmed to remove low-confidence base calls, and forward and reverse reads were aligned and assembled into a consensus sequence using BioEdit Sequence Alignment Editor (Version 7.2.5; Raleigh, NC, USA, 2013). Phylogenetic analyses were carried out using the Sequencing Analysis Software v5.4 (Applied Biosystems, Foster City, CA, USA) and MEGA X software (Version 10.0) [114] with an alignment created by the MUSCLE algorithm v3.8.31 [115]. Maximum Likelihood analysis was performed using the Kimura 2-parameter substitution model, with evolutionary rate variation among sites modelled using a gamma distribution with five discrete categories. The robustness of the tree topology was assessed using the bootstrap method with 100 replicates, and bootstrap support values were displayed at the corresponding nodes. All alignment sites, including positions containing gaps or missing data, were retained in the analysis. Tree searching was performed using the Nearest-Neighbor-Interchange (NNI) heuristic method, with the initial tree generated automatically using the NJ/BioNJ approach. For the phylogenetic analysis, we included strains preliminarily identified as *Bacillus* spp. members, together with two technical reference sequences, B110/23 and B111/23, which were included solely for phylogenetic comparison and were not part of the set of 93 bacterial isolates selected. In the generated dendrogram, we included the additional sequences of the *Bacillus*/*Priestia* species, which have already been published and uploaded to the NCBI database (NCBI; http://www.ncbi.nlm.nih.gov). The sequences of all strains were deposited in the National Centre for Biotechnology Information (NCBI; http://www.ncbi.nlm.nih.gov) [116] under the accession numbers displayed in Table A1, for the V6–V9 region.

### 4.4. Whole-Genome Sequencing and Genomic Characterization

To refine the preliminary 16S rRNA-based taxonomic assignment and perform whole-genome characterization of the selected strains (Table 1), whole-genome sequencing was conducted using the Illumina^®^ MiSeq v3 platform. Because partial 16S rRNA gene sequences may provide insufficient resolution for closely related bacterial taxa, genome-based identification was considered the more precise taxonomic assignment in subsequent analyses. The quality and quantity of isolated DNA from the selected strains were measured using a NanoDrop 2000 (Thermo Fisher Scientific, Waltham, WI, USA) and a Quantus fluorometer (Promega, Madison, WI, USA) with QuantusFluor DNA dsDNA reagent. Obtained DNA samples were then diluted with PCR-grade water to obtain equal concentrations ranging from 100 to 500 ng in a total volume of 30 µL. Library preparation was performed according to the Illumina DNA Prep Reference Guide (Illumina, San Diego, CA, USA), with assumptions similar to those of Price et al. [117], but modified by us. Prepared DNA was tagmented at 55 °C and then post-tagmented and cleaned up. Amplification was performed using a PCR program, with the cycle repeated eight times. The quality and quantity of received libraries were measured using D1000 ScreenTape and TapeStation 4150 system (Agilent Technologies, Santa Clara, CA, USA), and Quantus fluorometer (Promega, Madison, WI, USA), successively. Using the Illumina MiSeq system and the MiSeq Reagent Kit v3 (600-cycle) (Illumina, Inc., San Diego, CA, USA), samples were sequenced. Raw reads were base-called and demultiplexed with on-board primary analysis software. Cutadapt v4.4 was used to quality-filter the raw reads and trim adapter and primer sequences [118]. Then, the de novo assembly of the genome was conducted using the SPAdes genome assembler [119]. Assembly quality was assessed with QUAST v5.2, and gene prediction and annotation were performed with Prokka v1.14.6. The functional annotation of the genomes was performed with eggNOG-mapper v6.0 [120]. Quality and completeness were evaluated using QUAST v5.2 [121]. For identification of the products, in terms of antimicrobial resistance and mobile genetic elements, Comprehensive Antibiotic Resistance Database–Resistance Gene Identifier (CARD RGI v6.0) [122] and mobileOG-db beatrix-1.6 [123] were used. Sequence comparison and alignment were executed with Basic Local Alignment Search Tool [124] and National Center for Biotechnology Information resources [125]. Functional gene screening was performed manually following genome annotation. Gene names and predicted functions were verified using KEGG Release 101.0 and available genome annotations, and their biological relevance was assessed based on the peer-reviewed literature. Genes were assigned to functional categories when their annotated functions showed a literature-supported direct or indirect association with a given process; therefore, categories were not mutually exclusive and individual genes could contribute to more than one category. The selected functions represented mechanisms relevant to plant-associated inoculants, including plant growth and development, tissue colonization, holobiont interactions, nutrient uptake, abiotic and biotic stress resistance, and safety-related traits. Gene counts were interpreted as genomic functional potential rather than direct evidence of phenotype. For strain prioritization, this potential was considered together with taxonomic identity and safety-related genomic features, particularly antimicrobial-resistance-associated determinants; strains with an unacceptable safety profile were not considered promising application candidates. A comprehensive in silico analysis was performed to identify antimicrobial resistance genes and mobile genetic elements (MGEs) within the bacterial genomes. Antimicrobial resistance genes, biocide and metal resistance genes, and relevant mutations were screened with RGI (Resistance Gene Identifier) using CARD (Comprehensive Antibiotic Resistance Database) v3.2.x [122] and then further analyzed using AMRFinderPlus v3.11.26 [126]. Contig classification into chromosomal and plasmid sequences, along with replicon typing, was performed using the mob_recon module from the MOB-suite v3.1.0 [127]. Integrons were detected with Integron_Finder v2.0 [128], while plasmid and viral sequences, including prophages, were identified using geNomad [129]. The search for MGEs was supplemented with MobileElementFinder v1.1.2 [130] and detection of insertion sequences (IS) using the ISfinder database [131] via ABRicate v1.4.0 [132]. Finally, the precise identification of prophage islands was further enhanced using PIDE v1.1 [133]. Following the consolidation of all individual sample outputs, the parsed results were aggregated into a comprehensive dataset that enabled analyses across the sequenced isolates. The raw DNA sequencing data generated in the study are available in the Sequence Read Archive (SRA) database at the National Center for Biotechnology Information (NCBI) https://www.ncbi.nlm.nih.gov/, with accession number PRJNA1469014.

### 4.5. Phenotypic Characterization Using Biolog GEN III

Phenotypic characterization of metabolic activity and chemical sensitivity of selected strains was performed using the BIOLOG MicroStation System (Biolog Inc., Hayward, CA, USA) in combination with GENIII MicroPlates, according to the manufacturer’s instructions. This system enables high-throughput profiling of microbial phenotypes based on patterns of carbon source utilization and responses to chemical stressors. GEN III MicroPlates comprise 96 wells, including 72 substrates for carbon utilization and 23 assays assessing sensitivity to various inhibitory compounds, allowing for comprehensive metabolic fingerprinting [134]. In this study, the term “substrate” refers to an individual carbon source present in a GEN III MicroPlate well and available for metabolic utilization by the tested strain. Six strains (B56/23, B64/23, B80/23, B99/23, B105/23, B107/23), selected based on prior genomic and molecular analyses, were cultured on BC medium and incubated for 24 h at 28 °C. The strains were selected to represent the most relevant candidates emerging from the previous molecular and genomic screening, taking into account taxonomic identity, the presence of plant-associated functional traits, overall genomic potential, and safety-related features. Subsequently, bacterial biomass from each strain was harvested and suspended in inoculating fluid IF-A (Biolog Inc., Hayward, CA, USA). The cell density was adjusted to a transmittance of 95–98% using a turbidimeter (Biolog Inc., Hayward, CA, USA), ensuring standardized inoculum conditions. Prepared suspensions were dispensed into GENIII MicroPlates (100 μL per well), using a multichannel pipette. For each strain, three independent plates were used as biological replicates. Plates were incubated in the OmniLog system (Biolog Inc., Hayward, CA, USA) at 26 °C for 72 h. Colorimetric measurements were recorded at defined time points (0, 4, 8, 12, 20, 24, 28, 32, 35, 44, 48, 52, 56, 59, and 72 h) using a MicroStation plate reader (ELX808BLG, Biolog Inc., Hayward, CA, USA). Absorbance was measured at two wavelengths: 590 nm, which reflects respiratory activity, and 750 nm, corresponding to biomass production [2]. Raw data were collected using MicroStation™ system with MicroLog™ software v1.4 (Biolog Inc., Hayward, CA, USA) and subsequently processed and statistically analysed using Statistica™ software (TIBCO Software Inc., version 13.3). Metabolic and chemical sensitivity profiles were evaluated using three parameters: absorbance at 590 nm, representing respiratory activity associated with substrate utilization; absorbance at 750 nm, reflecting biomass production; and the Substrate Stress Index (SST), calculated as:

$$\mathrm{SST}\;=\;\frac{A590}{A750}$$

where A590 and A750 represent absorbance measured at 590 and 750 nm, respectively. Higher SST values indicate greater respiratory activity relative to biomass production, whereas lower values indicate proportionally greater biomass production relative to respiratory activity. Carbon-source utilization and chemical sensitivity were analysed separately. For carbon-source utilization, responses in wells containing the 72 carbon substrates were compared based on A590, A750, and SST values. Chemical sensitivity was evaluated analogously for the 23 wells containing inhibitory compounds, allowing comparison of strain-specific responses to chemical stressors. For visualization, heatmaps were generated from the final 72-h measurements, selected to represent the endpoint metabolic profiles after the major changes in substrate utilization had stabilized. Separate heatmaps were generated for A590, A750, and SST for both carbon-source utilization and chemical sensitivity assays. We chose the last reading (after 72 h of incubation) to maintain the integrity of the substrate utilization results. Statistical analyses were performed using Statistica™ software (TIBCO Software Inc., version 13.3) and, where indicated, additional analytical workflows. The statistical approach was selected separately for each data type. Endpoint measurements obtained after 72 h were analysed separately for respiratory activity at 590 nm, biomass-associated responses at 750 nm, and the Substrate Stress Index (SST). Data normality was assessed using the Shapiro–Wilk test and homogeneity of variance using Levene’s test. Depending on the distribution and variance structure, appropriate parametric or non-parametric comparisons were applied. Hierarchical cluster analysis used for heatmap visualization was performed separately using Euclidean distance and Ward’s linkage method. Time-resolved BIOLOG data were additionally analysed using kinetic parameters, including area under the curve (AUC), peak response, time to peak, slope, and growth rate. AUC values were compared among strains using the Kruskal–Wallis test followed by Dunn’s pairwise post hoc test. *p*-values for multiple comparisons were adjusted using the Benjamini–Hochberg false discovery rate (FDR) procedure, with adjusted *p* < 0.05 considered statistically significant. Principal component analysis (PCA) was used to assess multivariate differences among strain-specific BIOLOG profiles. All graphical visualizations were generated using R software, version 4.6.0 (R Core Team, Vienna, Austria) environment based on the corresponding statistical analysis outputs.

### 4.6. Transcriptomic Analysis by RNA-Seq

To examine gene expression profiles and transcriptome differences among the 2 selected strains (Table A1), we isolated total RNA and performed next-generation sequencing of the transcriptomes using the Illumina^®^ MiSeq platform. Overnight bacterial cultures incubated at 23 °C in BC medium were centrifuged to obtain the bacterial pellets, which were subjected to enzymatic lysis using the lysozyme (5 mg mL^−1^) for 30 min at room temperature to guarantee effective breakdown of the Gram-positive cell wall. RNA was isolated with GeneMATRIX Universal RNA Purification Kit (EURx, Gdańsk, Poland) according to the manufacturer’s protocol. The residual genomic DNA was removed with on-column DNase I treatment. The quantity and quality of the obtained RNA were measured with RNA ScreenTape and TapeStation 4150 system (Agilent Technologies, Santa Clara, CA, USA), and NanoDrop 2000 spectrophotometer (Thermo Fisher Scientific, Waltham, MA, USA). Library preparation followed the Illumina Stranded mRNA Prep (Illumina, San Diego, CA, USA), with slight modifications. Initially, messenger RNA was purified and fragmented, leading to first-strand cDNA synthesis and second-strand cDNA synthesis. Obtained fragments were adenylated at the 3′ ends. This step enabled the implementation of Illumina DNA/RNA UD Indexes ligation to the libraries. After this step, the cleaning up was performed using CleanNGS magnetic beads (CleanNA, Gouda, The Netherlands). Subsequently, indexed libraries were amplified according to the program: 95 °C for 3 min; 12 cycles of 95 °C for 30 s, 55 °C for 30 s, and 72 °C for 60 s, followed by a final extension at 72 °C for 5 min. After this step, the second magnetic bead cleanup was performed. The quantity and quality of the libraries obtained were measured using the D1000 ScreenTape and TapeStation 4150 systems (Agilent Technologies, Santa Clara, CA, USA) and a Quantus fluorometer (Promega, Madison, WI, USA). Thereafter, the libraries were normalized to 4 nmol L^−1^, pooled, and denatured. Sequencing was performed with the Illumina^®^ MiSeq v3 platform using the MiSeq Reagent Kit v3 (150-cycle) in the paired-end mode. Raw reads were basecalled and demultiplexed with on-board primary analysis software. Trimming and quality filtering of the raw paired-end reads were performed using Cutadapt v4.4 [118]. Then the reads were aligned to previously sequenced genomes with Spliced Transcripts Alignment to a Reference (STAR) v2.7.10b [135] and quantified with Salmon v1.9.0 [136]. A transcriptomic analysis was performed to characterize gene expression and functional profiles across the two studied strains. Transcript assemblies were generated from prokka v1.14.6 genome annotations using GffRead v0.12.7 [132,137], and transcript-level quantification was performed with Salmon v1.9.0 [136]. Orthologous gene groups were identified across strains using OrthoFinder v1.0 [138], and functional annotation of the orthogroup representatives was performed with eggNOG-mapper v2.1.9 [120,139]. Custom Python, version 3.14.5 (Python Software Foundation, Wilmington, DE, USA) scripts were employed to aggregate Salmon quantifications to the gene level and to extract representative sequences from each orthogroup for downstream analyses. Differential expression analysis and functional enrichment were performed using R software environment v4.2.3. Gene-level count data aggregated into orthogroups were analyzed to identify differentially expressed orthogroups between the compared strains using the DESeq2 package v1.38.3 [140]. Data manipulation, transformation, and graphical visualizations were carried out with the Tidyverse suite v1.3.2 [141], with additional plotting functions from ggplot2 v3.4.0 [142]. Functional annotations, including KEGG pathway assignments derived from eggNOG-mapper outputs, were processed for pathway-level aggregation. Official KEGG pathway names and identifiers were retrieved using the KEGGREST package v1.38.0 [143], enabling the construction of pathway-specific expression profiles and the identification of significantly modulated metabolic routes between the strains.

The raw RNA sequencing data generated in the study are available in the Sequence Read Archive (SRA) database at the National Center for Biotechnology Information (NCBI) https://www.ncbi.nlm.nih.gov/, with the accession number: PRJNA1469014.

### 4.7. Morphological Analysis by Holotomography

Six selected strains (B56/23, B64/23, B80/23, B99/23, B105/23, B107/23) were precultured on BC medium and incubated for 24 h at 28 °C. Bacterial biomass from each strain was collected and suspended in 1.5 mL of sterile 0.85% sodium chloride solution, then vortexed to obtain a homogeneous cell suspension. A 500 µL volume of each suspension was transferred to imaging dishes (Ibidi GmbH, Gräfelfing, Germany). Bacterial strains were analysed with Nanolive 3D Cell Explorer-fluorescence holotomography microscope (Nanolive, Tolochenaz, Switzerland), using label-free, high-resolution visualization of cellular morphology. For each strain, multiple fields of view were examined to account for within-sample morphological variability. Cells were visualized in the label-free mode based on differences in refractive index, and representative 2D and 3D reconstructions were generated using STEVE software, v.2.6 (Nanolive, Tolochenaz, Switzerland). Digital image processing and cellular measurements were performed using Fiji (version Java 8) [144]. To ensure accurate cell quantification, 8-bit grayscale images were pre-processed using a Rolling Ball background subtraction (radius = 20 pixels). Segmentation of the bacteria was performed by thresholding method to generate a binary mask. Gaussian blur (σ = 1.0 pixel) was applied to prevent over-splitting during the automated Watershed separation algorithm. Parameters were quantified using the Analyze Particles tool, with an area threshold filter. Object shapes were fitted to best-fit ellipses to extract individual cell length and width. Image analysis included qualitative assessment of cell shape, relative size, elongation, cellular arrangement (individual cells, chains, clusters, or multilayered structures), and intracellular refractive-index heterogeneity. Representative images were selected only from fields containing clearly resolved, non-overlapping cells and without obvious imaging artefacts. Holotomographic imaging was used for qualitative comparative characterization of cellular morphology rather than for quantitative morphometric analysis. Approximate cell dimensions were estimated from the spatial scale provided by the imaging software and were used only as descriptive morphological characteristics. The purpose of this analysis was to provide an additional phenotypic description of the selected strains and to identify visually distinct morphological features complementing their molecular and genomic characterization.

For the purposes of this study, promising strains were defined as isolates combining a broad plant-associated functional potential with favourable characteristics across the analysed genomic, metabolic, morphological, and, where available, transcriptomic datasets, while maintaining an acceptable genomic safety profile. Safety was treated as an essential selection criterion; therefore, potentially beneficial functional traits alone were not considered sufficient to support application potential.

## 5. Conclusions

From over a hundred isolated strains, the aim was to identify a candidate with the most promising functional and safety-related characteristics for subsequent validation in microgreen systems. Integration of genomic, metabolic, morphological, and transcriptomic data identified strain B107/23 as the most promising candidate among the analyzed isolates. Its combination of metabolic flexibility, a balanced metabolic profile and the presence of stress-associated functional traits, and potential for microbial interaction suggests strong adaptability to complex environments. The presence of traits potentially associated with stress adaptation, microbial interactions, and competitive fitness supports the selection of this strain as a candidate for further evaluation as a bioinoculant. In contrast, isolates identified as *K. michiganensis* were excluded from further consideration as potential bioinoculants because of their opportunistic pathogenic status and associated safety concerns. Moreover, the comparatively low abundance of antibiotic resistance genes in B107/23 further supports its prioritization for subsequent safety assessment and application-oriented studies. These findings are consistent with the broad biotechnological and agricultural potential reported for *B. megaterium* and provide a basis for further in planta validation of B107/23 in microgreen systems, including evaluation of its effects on plant stress responses, microbiome structure, and post-harvest quality.

## Figures and Tables

**Figure 1:**
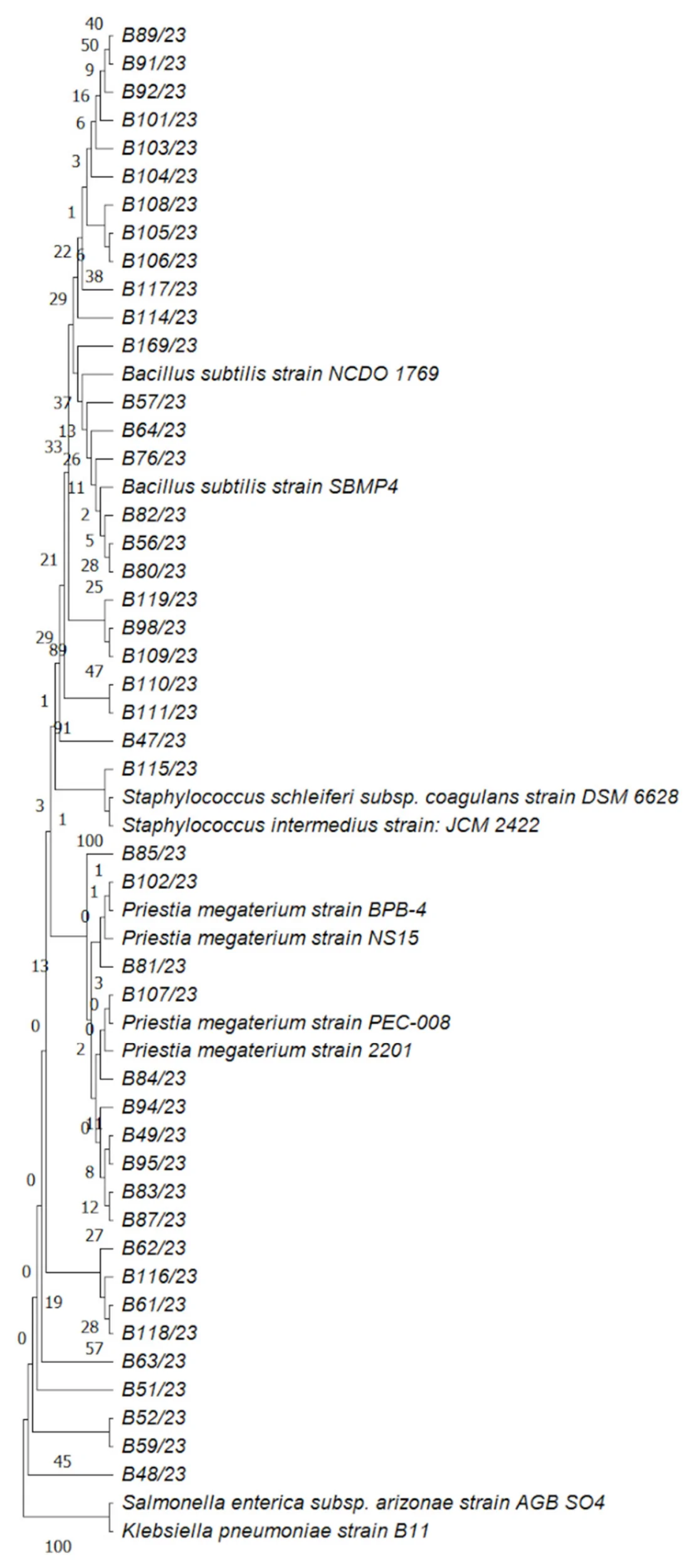
Phylogenetic tree based on the V6-V9 region of 16S rRNA gene sequences of *Bacillus*/*Priestia* spp. isolates.

**Figure 2:**
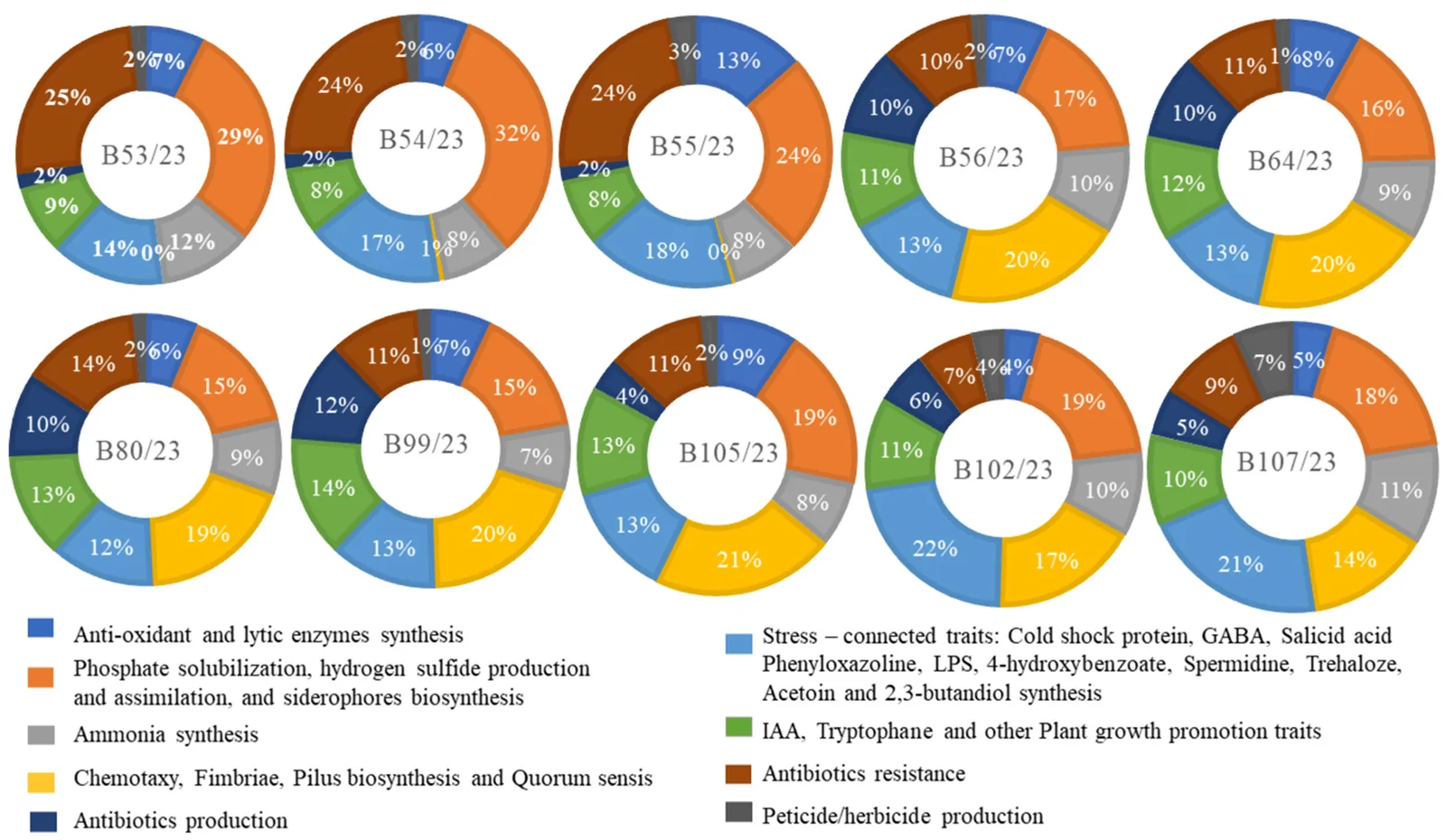
Comparative distribution of functional gene categories in the genomes of the examined strains. The analysed categories included genes associated with antioxidative and lytic enzyme synthesis, nutrient acquisition (phosphate solubilization, hydrogen sulfide production and assimilation, siderophore biosynthesis, and ammonia synthesis), chemotaxis, fimbriae and pilus biosynthesis, quorum sensing, stress-related traits, plant growth-promoting functions, antibiotic biosynthesis and resistance, and pesticide/herbicide resistance.

**Figure 3:**
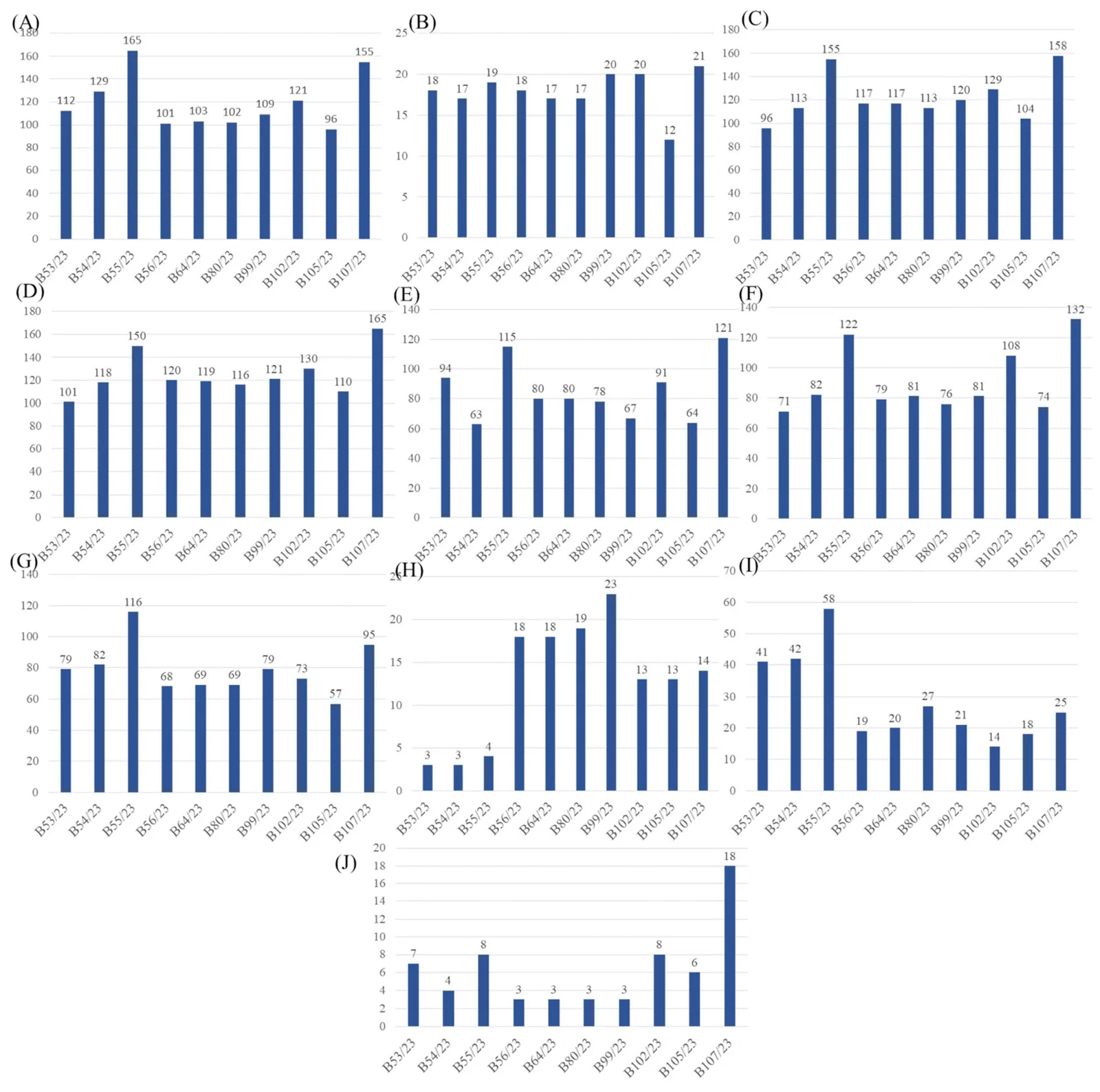
The comparative analysis of the examined strains’ genomes by function. Several identified genes contribute to the promotion and enhancement of the processes of (**A**)—Plants’ growth, (**B**)—Developmental processes within the plant, (**C**)—Plants’ tissue colonization, (**D**)—interactions within the holobiont, (**E**)—Nutrient uptake, (**F**)—Abiotic stress resistance, (**G**)—Biotic stress resistance. Genes directly connected to: (**H**)—Antibiotic production, (**I**)—Antibiotic resistance, (**J**)—Pesticide/herbicide resistance.

**Figure 4:**
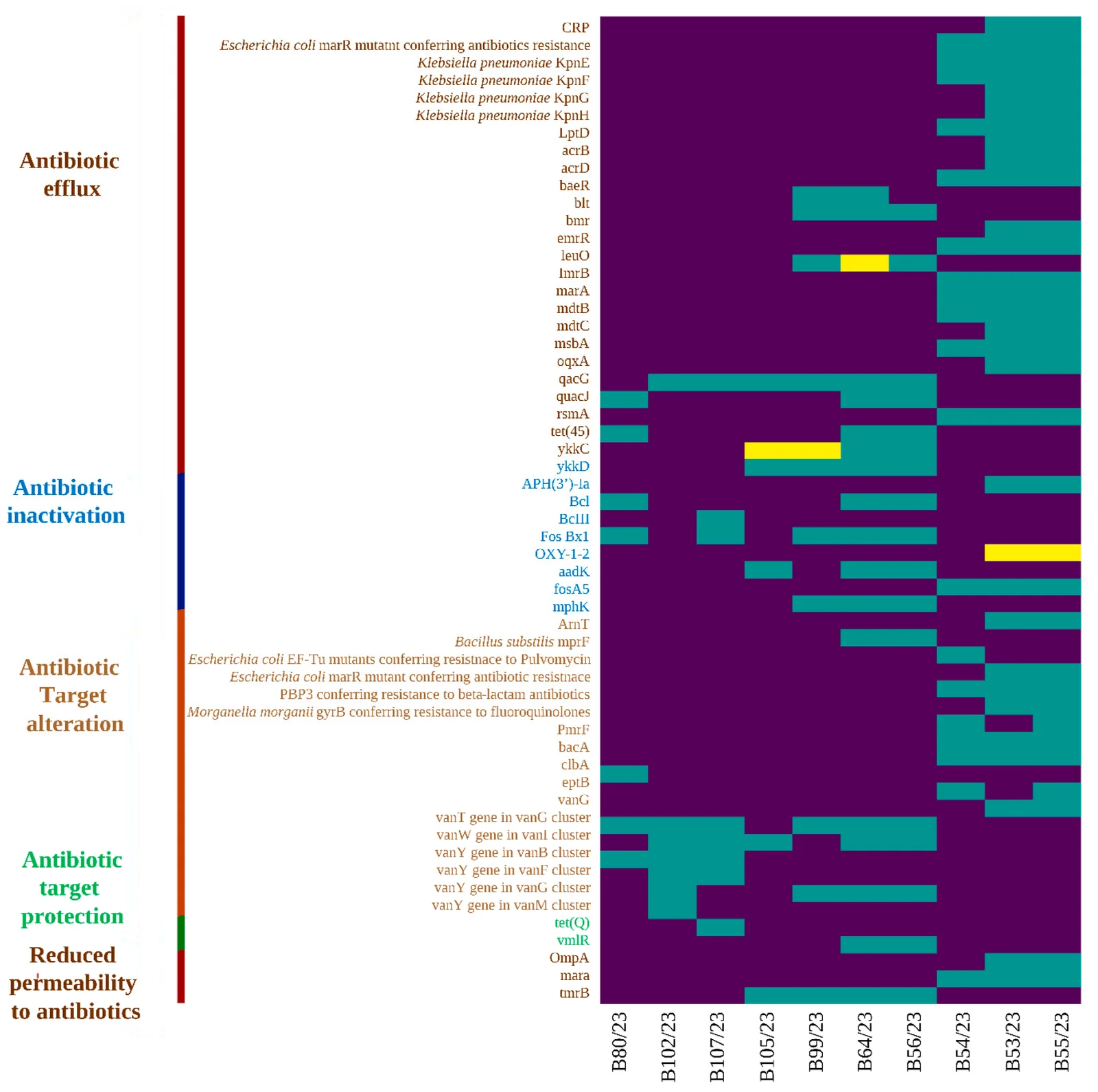
Heatmap exhibiting the presence of the antibiotic resistance genes across the examined strains. Colours indicate confidence in detection based on sequence homology.

**Figure 5:**
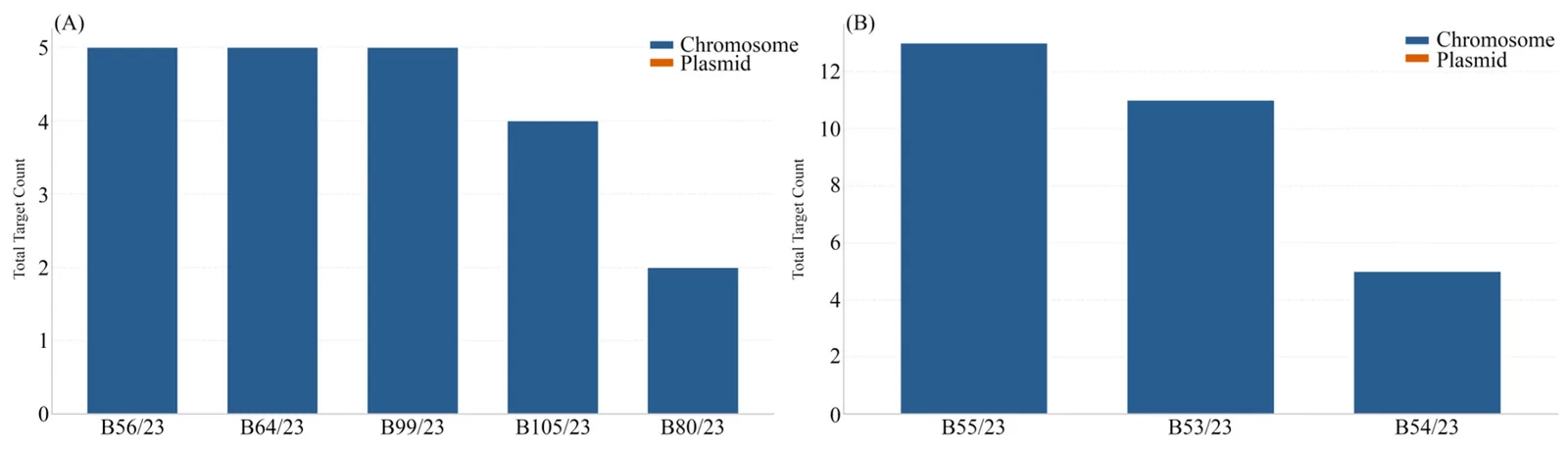
Genomic localization of antimicrobial resistance (AMR)-associated targets in the examined strains. Bar plots show the number of AMR-associated genes and resistance-associated mutations assigned to chromosomal or plasmid regions in *Bacillus*/*Priestia* strains (**A**) and *K. michiganensis* strains (**B**). Total target count represents the combined number of detected AMR-associated genes and mutations for each strain. The orange bars are not visible in the figure, indicating that no plasmid-associated mutations were identified.

**Figure 6:**
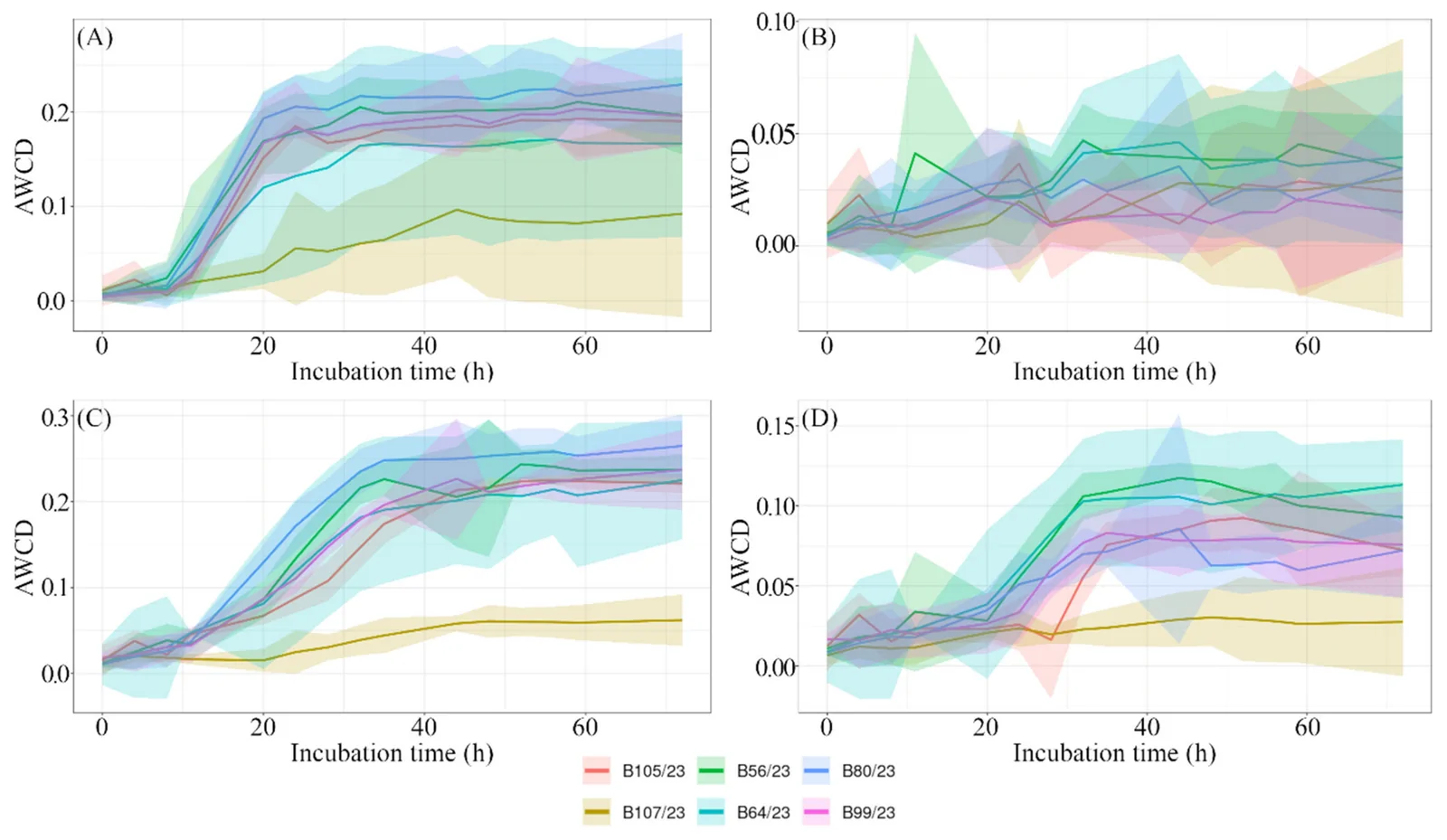
Time-resolved AWCD kinetic profiles of the examined strains obtained from BIOLOG: metabolic activity at 590 nm (**A**) and 750 nm (**B**), and chemical sensitivity at 590 nm (**C**) and 750 nm (**D**).

**Figure 7:**
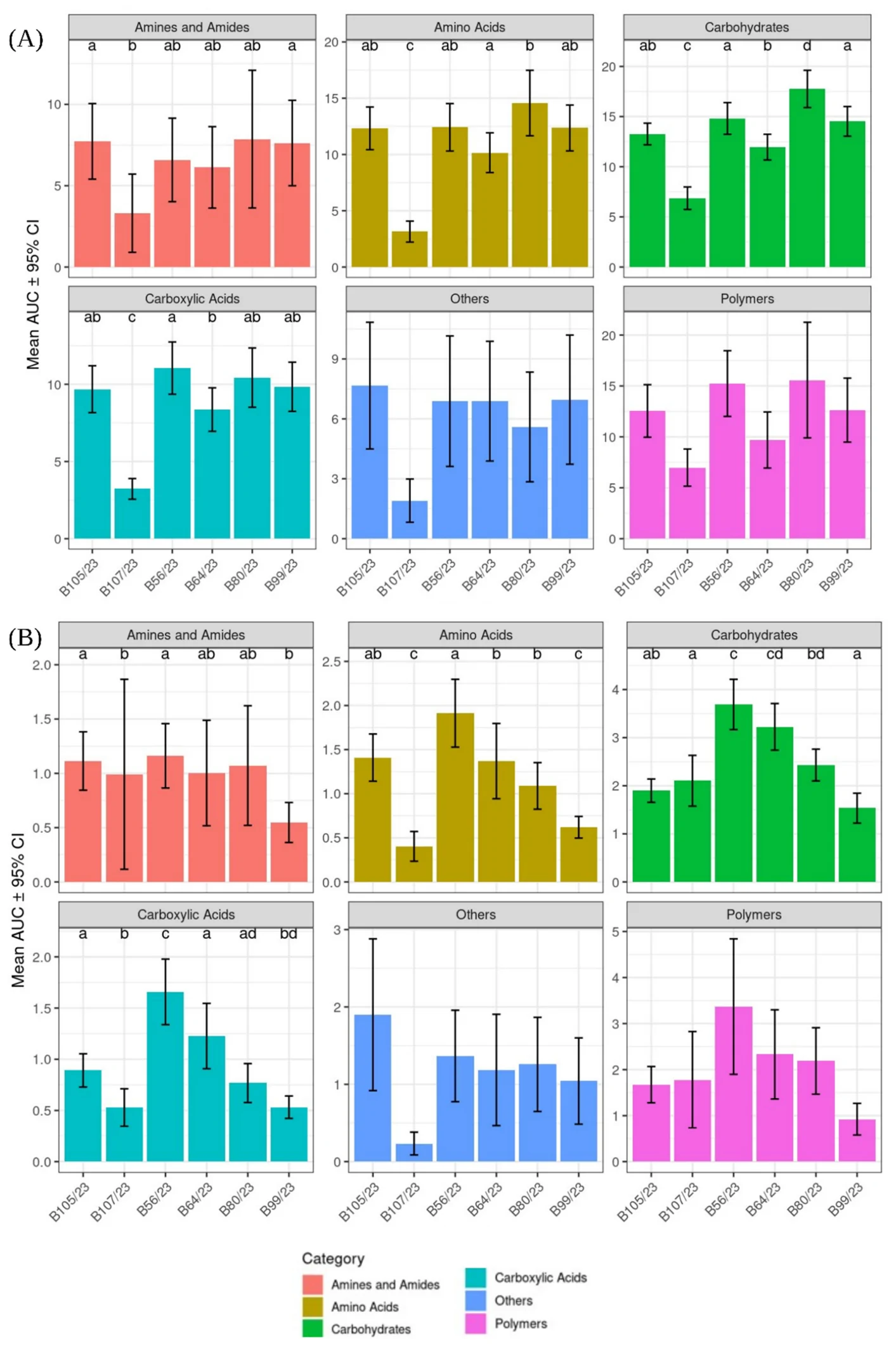
Area under the curve (AUC) of substrate utilization by the examined strains across individual substrate categories. AUC values are presented for measurements at 590 nm (**A**) and 750 nm (**B**). Different letters (a–d) indicate statistically significant differences between groups.

**Figure 8:**
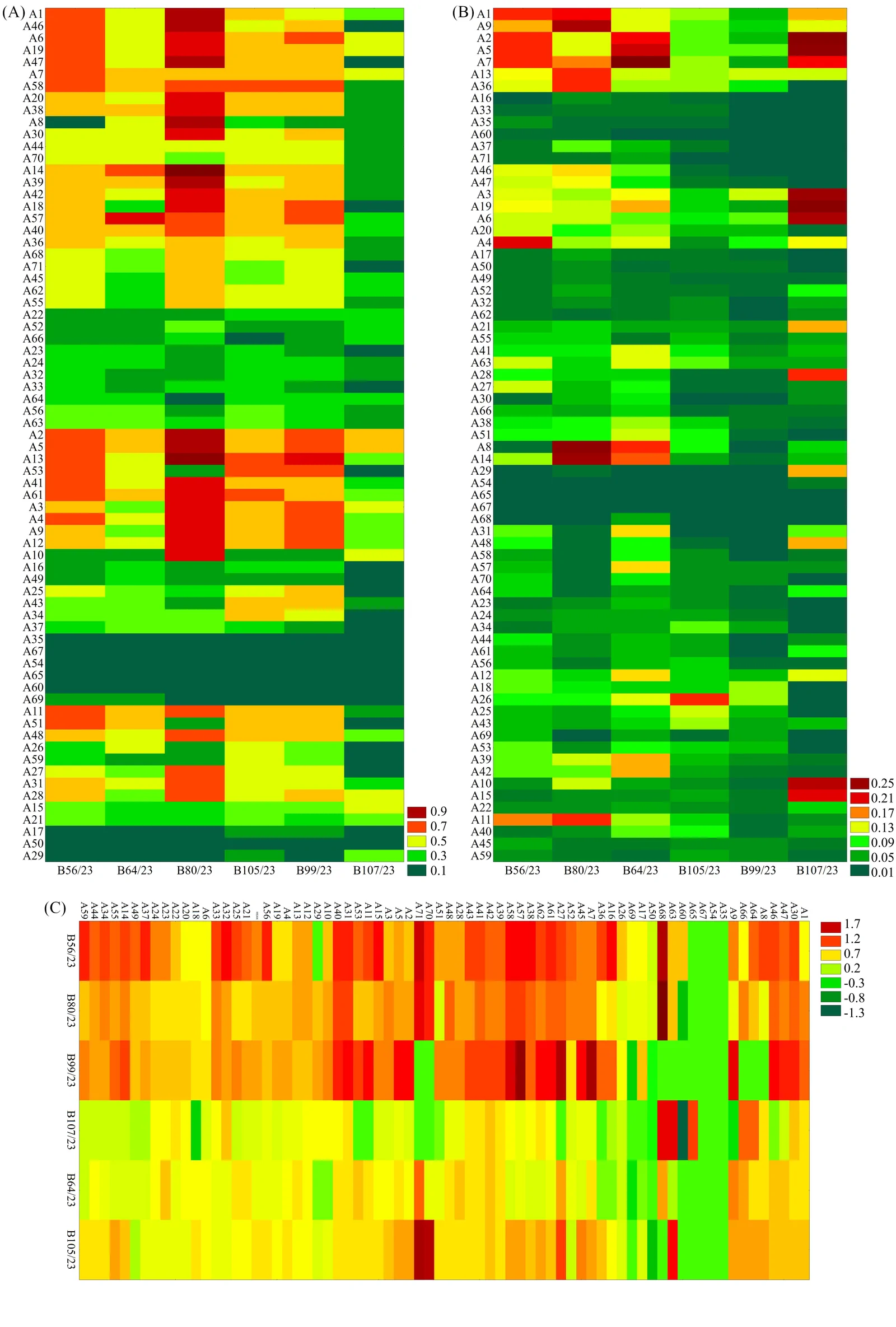
Heatmaps displaying the utilization of specific substrates for respiratory reactions (**A**) and biomass production (**B**) after 72 h of incubation; heatmap of the substrate stress index displayed for the examined strains (**C**).

**Figure 9:**
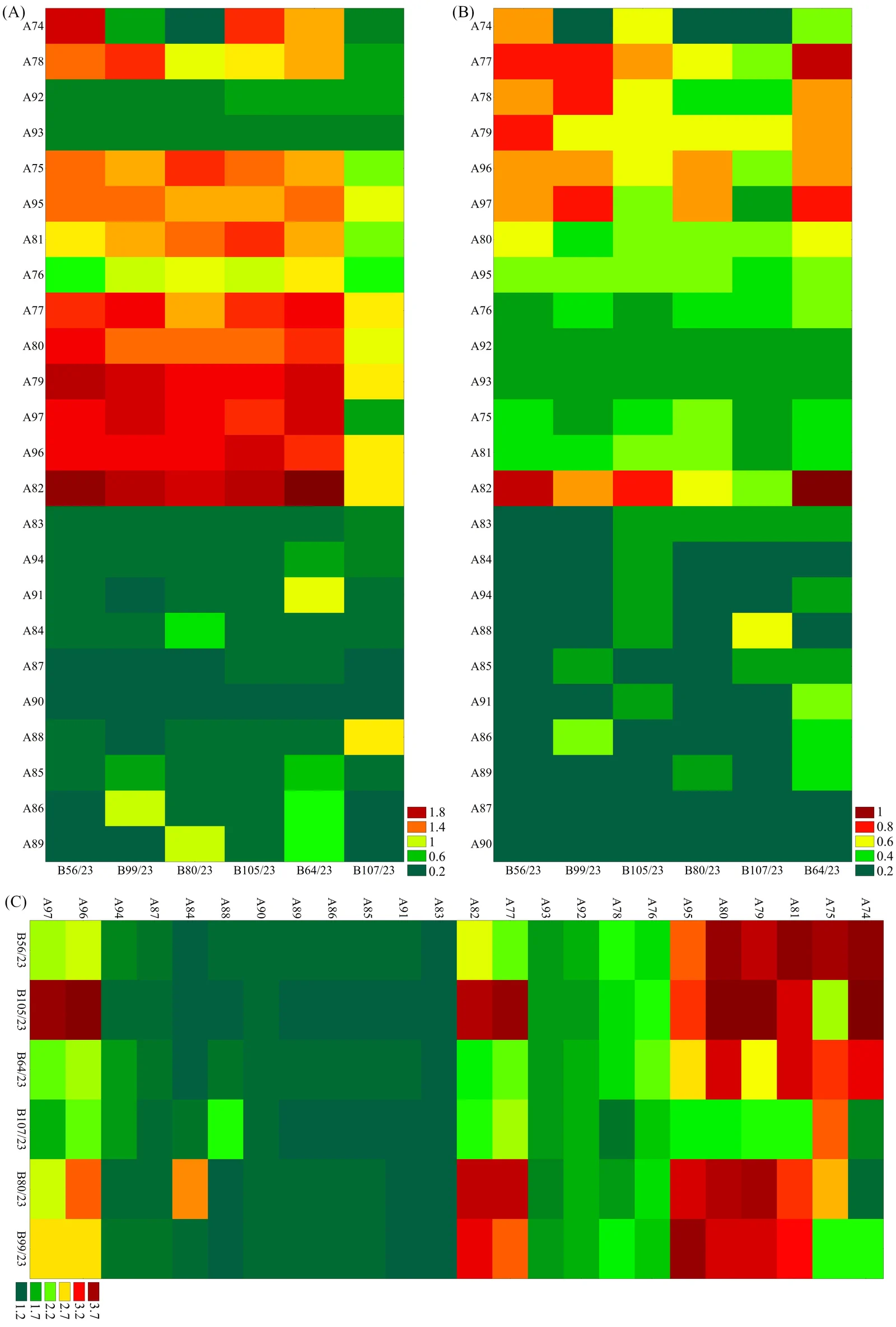
Heatmaps displaying the inhibition of respiratory reactions (**A**) and biomass production (**B**) after 72 h of incubation by specific substrates; heatmap of the substrate stress index displayed in the presence of the specific substrates for the examined strains (**C**).

**Figure 10:**
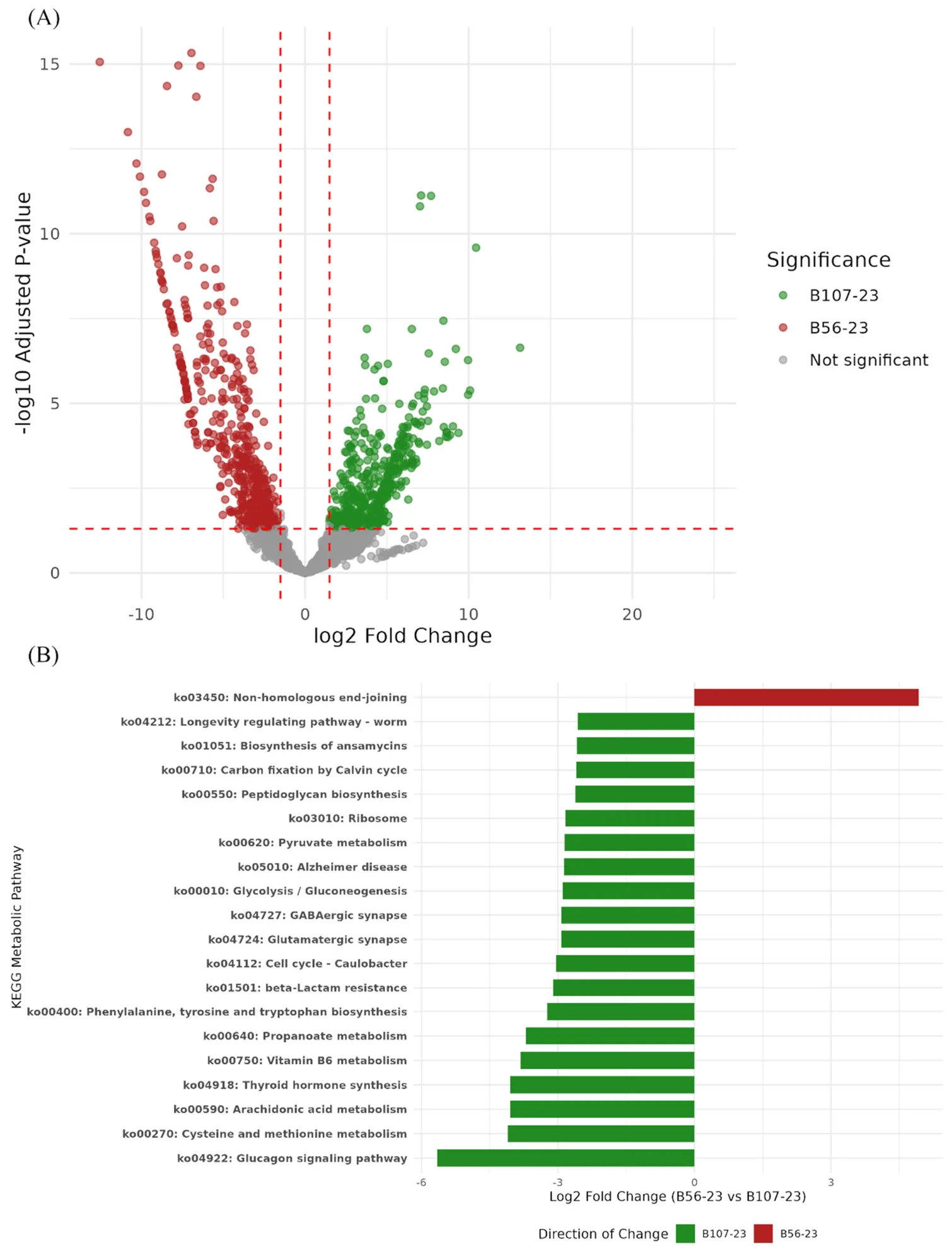
Differential transcriptomic comparison of B56/23 and B107/23. (**A**) Volcano plot of differentially expressed orthologous groups. (**B**) Top 20 KEGG pathways with the largest expression differences between the strains.

**Figure 11:**
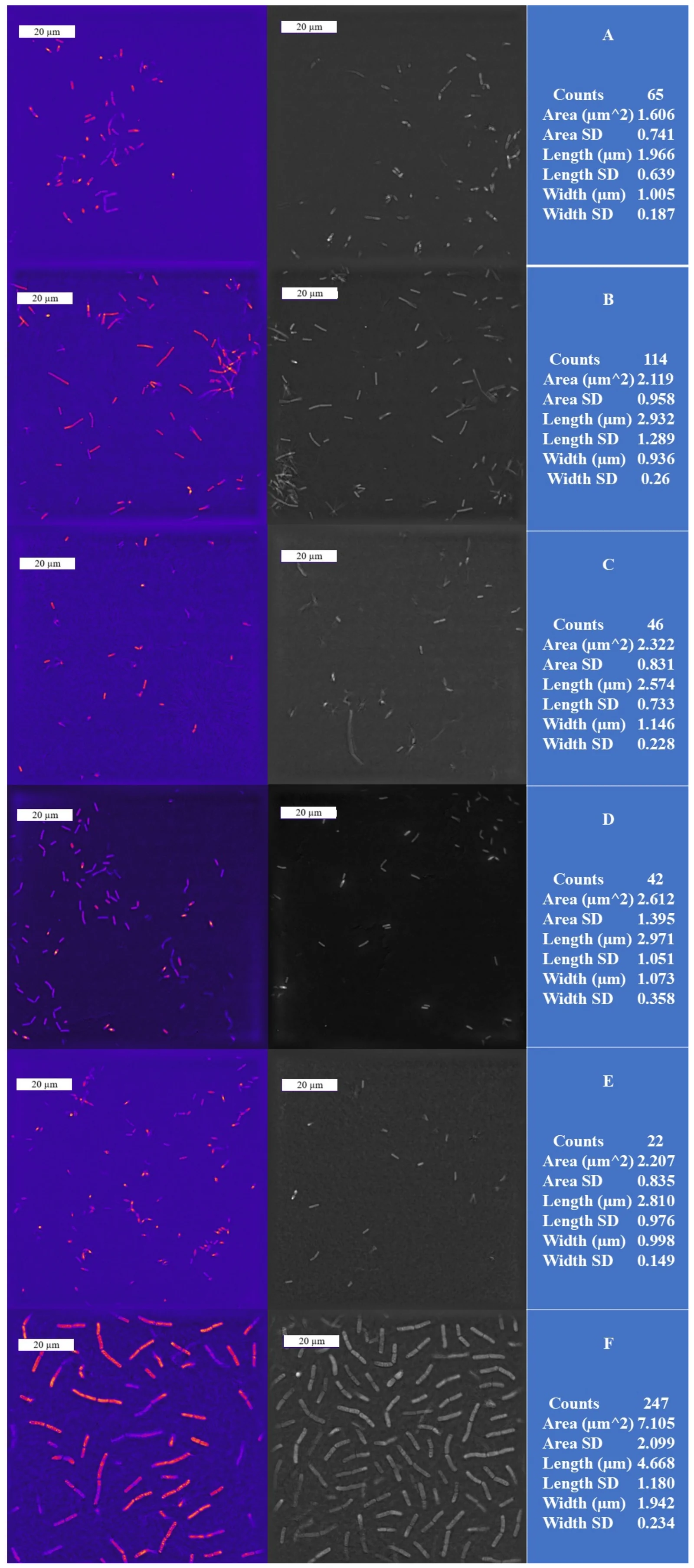
Images of 2D pictures of strains B56/23 (**A**), B64/23 (**B**), B80/23 (**C**), B99/23 (**D**), B105/23 (**E**), and B107/23 (**F**) taken via NanoLive Holotomography Microscopy. The scale bars indicate 20 µm.

**Table 1:** General characterization of the genomes of investigated strains.

Strain	BLAST Final Identification	Genome Size	Total Gene Count	Coding Sequences (CDS)	Automatically Annotated Gene Number
B53/23	*Klebsiella michiganensis*	6,179,014	5746	5663	4120
B54/23	*Klebsiella michiganensis*	6,449,535	7679	7617	2431
B55/23	*Klebsiella michiganensis*	6,232,323	5797	5711	4209
B56/23	*Bacillus subtilis*	4,637,937	4752	4667	2762
B64/23	*Bacillus subtilis*	4,101,437	4108	4016	2756
B80/23	*Bacillus subtilis*	3,910,465	3819	3731	2546
B99/23	*Bacillus subtilis*	4,873,426	5003	4912	2166
B102/23	*Priestia megaterium*	8,156,527	9257	9134	2213
B105/23	*Bacillus subtilis*	4,837,141	7802	7735	1382
B107/23	*Priestia megaterium*	6,764,679	6804	6690	3690

**Figure A1:**
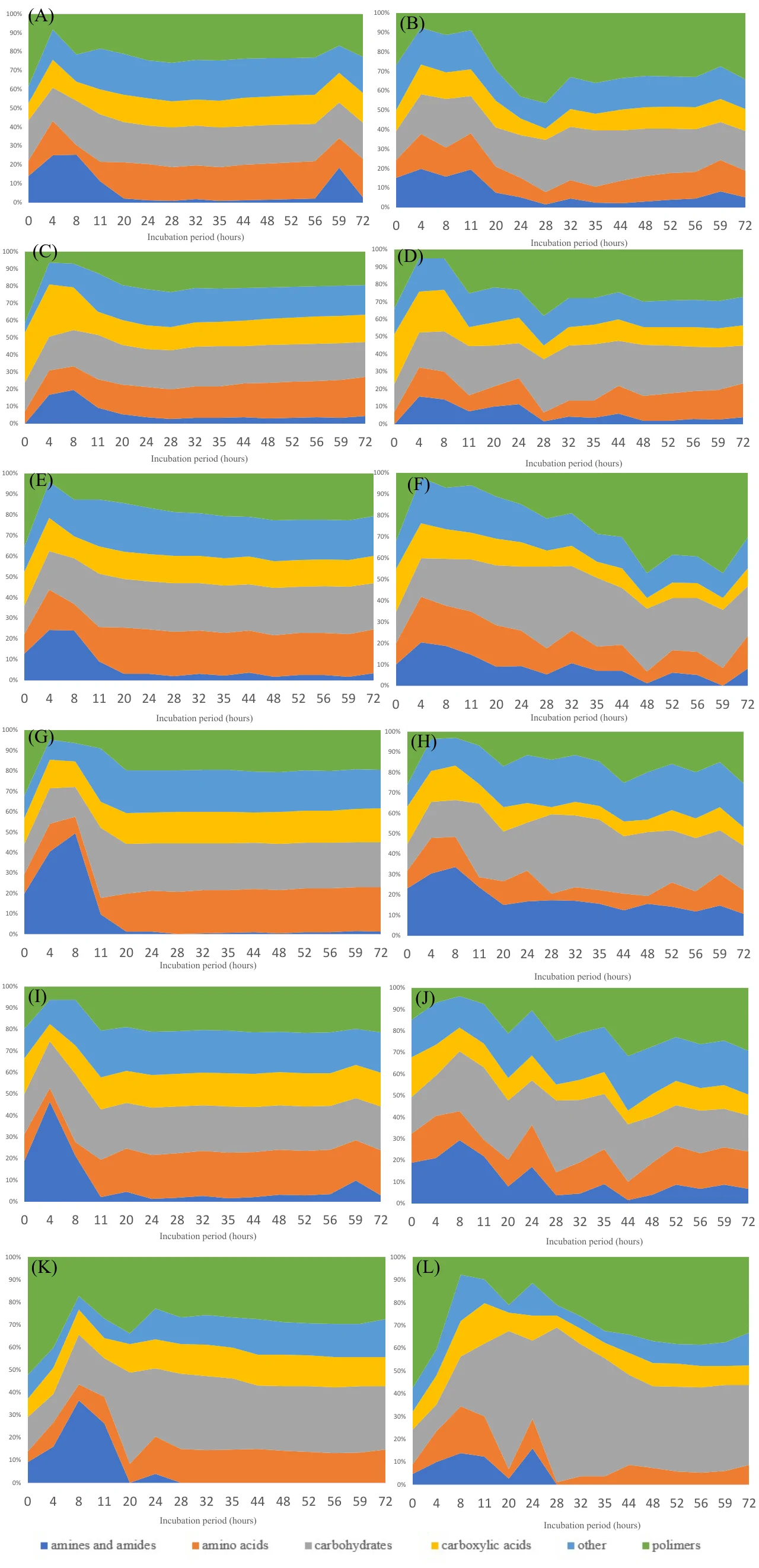
The figure presents the substrate utilization kinetics for both respiratory activity and biomass production of the examined strains, based on time-resolved absorbance profiles. The strains are presented sequentially as B56/23, B64/23, B80/23, B99/23, B105/23, and B107/23, with paired panels corresponding to measurements at 590 nm and 750 nm. Panels A–L therefore represent: (**A**,**B**), B56/23 (590 and 750 nm); (**C**,**D**), B64/23 (590 and 750 nm); (**E**,**F**), B80/23 (590 and 750 nm); (**G**,**H**), B99/23 (590 and 750 nm); (**I**,**J**), B105/23 (590 and 750 nm); and (**K**,**L**), B107/23 (590 and 750 nm). Absorbance at 590 nm reflects respiratory activity associated with substrate utilization, whereas absorbance at 750 nm represents biomass-associated turbidity.

**Figure A2:**
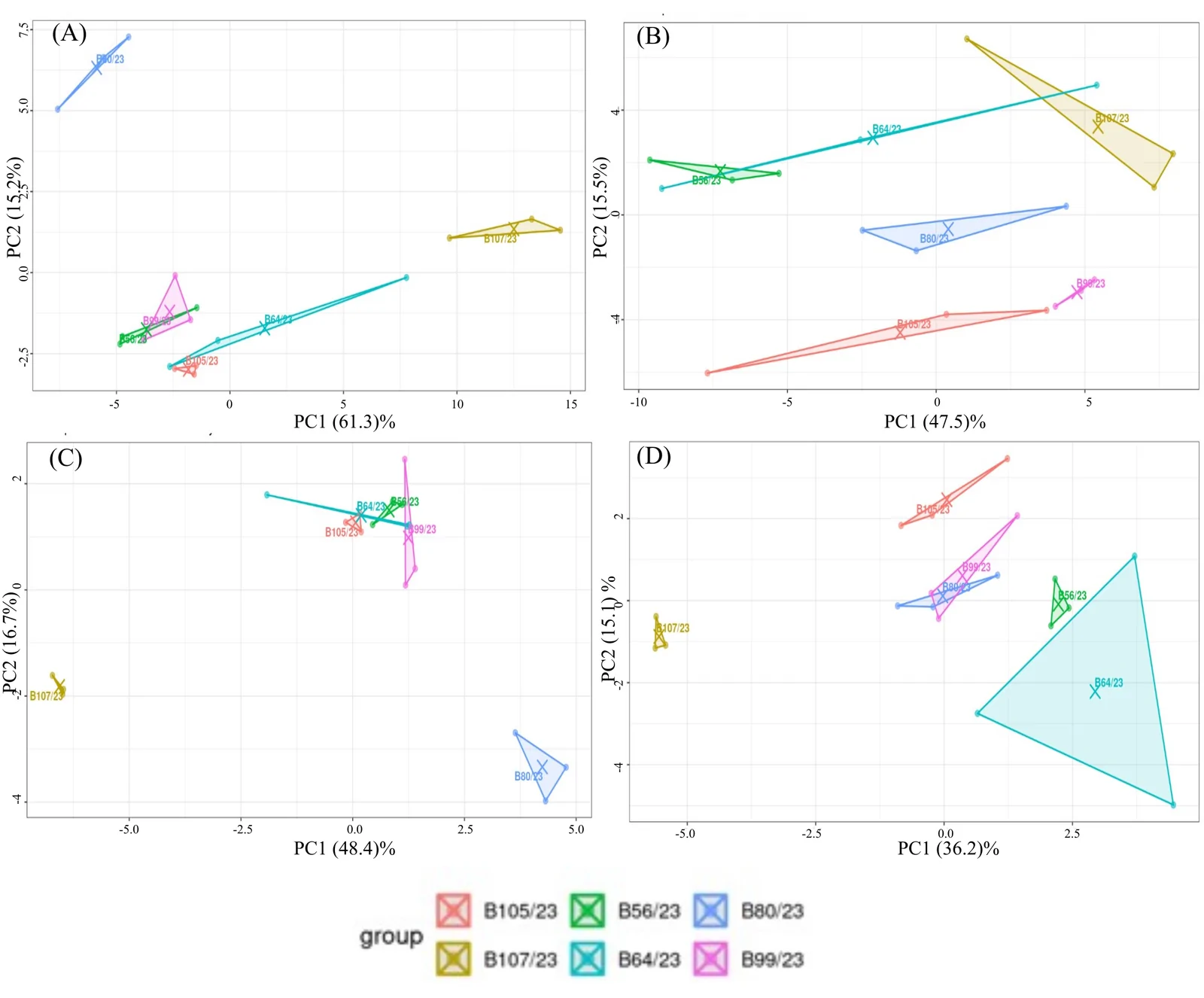
Principal component analysis (PCA) of BIOLOG profiles of the examined strains. Metabolic activity is shown at 590 nm (**A**) and 750 nm (**B**), whereas chemical sensitivity is shown at 590 nm (**C**) and 750 nm (**D**).

## Data Availability

The bacterial sequences generated in this study have been deposited in the NCBI nucleotide database under the following accession numbers, [PX720931–PX720984; PX720987-PX721025], and in the Sequence Read Archive (SRA) under the following accession number, [PRJNA1469014]. All other data supporting the findings of this study are included within the manuscript. Raw data are available from the corresponding author upon reasonable request.

## Abbreviations

The following abbreviations are used in this manuscript:

PGPR	Plant Growth-Promoting Rhizobacteria
PCR	Polymerase Chain Reaction
16S rRNA	16S ribosomal RNA
BLAST	Basic Local Alignment Search Tool

## Appendix A

**Table A1:** The table presents the results of preliminary identification using PCR with the comK gene marker and the endoglucanase marker, as well as from Sanger sequencing. The accession numbers of the obtained sequences are provided. The table also includes the sources of isolation of the individual strains.

Strain Number	Identification with the *ComK* Gene Marker	Identification with Endoglucanase Gene Marker	Identification with Sanger Sequencing	Isolation Source	Accession Number
B47/23	-	-	*Peribacillus* sp.	Homemade pickled cucumbers	PX720931
B48/23	-	-	*Bacillus* sp.	Homemade pickled cucumbers	PX720932
B49/23	-	-	*Priestia* sp.	Homemade pickled cucumbers	PX720933
B50/23	-	-	*Bacillus* sp.	Homemade pickled cucumbers	PX720934
B51/23	-	-	*Bacillus* sp.	Homemade pickled cucumbers	PX720935
B52/23	-	-	*Bacillus* sp.	Homemade pickled cucumbers	PX720936
B53/23	-	-	*Klebsiella* sp.	Homemade fresh-pickled cucumbers	PX720937
B54/23	-	-	*Klebsiella* sp.	Homemade fresh-pickled cucumbers	PX720938
B55/23	-	-	*Klebsiella* sp.	Homemade fresh-pickled cucumbers	PX720939
B56/23	-	+	*Bacillus subtilis*	Cow’s powdered milk from Siedlce	PX720940
B57/23	-	+	*Bacillus* sp.	Cow’s powdered milk from Siedlce	PX720941
B58/23	-	-	*Bacillus* sp.	Cow’s powdered milk from Siedlce	PX720942
B59/23	-	-	*Bacillus* sp.	Homemade pickled cucumbers	PX720943
B60/23	-	-	*Bacillus* sp.	Homemade pickled cucumbers	PX720944
B61/23	-	-	*Bacillus* sp.	Homemade pickled cucumbers	PX720945
B62/23	-	-	*Bacillus* sp.	Homemade pickled cucumbers	PX720946
B63/23	-	-	*Bacillus* sp.	Homemade pickled cucumbers	PX720947
B64/23	-	+	*Bacillus subtilis*	Cow’s powdered milk from Krasnystaw	PX720948
B65/23	-	-	*Bacillus* sp.	Homemade pickled cucumbers	PX720949
B75/23	-	-	*Priestia* sp.	Pickled radish from Zakiszony	PX720950
B76/23	-	-	*Bacillus* sp.	Pickled radish from Zakiszony	PX720951
B77/23	-	-	*Priestia* sp.	Pickled radish from Zakiszony	PX720952
B78/23	-	-	*Lactiplantibacillus* sp.	Pickled radish from Zakiszony	PX720953
B79/23	-	-	*Lysinibacillus* sp.	Pickled radish with thyme from Sątyrz	PX720954
B80/23	-	+	*Bacillus* sp.	Pickled radish with thyme from Sątyrz	PX720955
B81/23	-	-	*Priestia* sp.	Pickled radish with thyme from Sątyrz	PX720956
B82/23	-	-	*Bacillus* sp.	Pickled radish with thyme from Sątyrz	PX720957
B83/23	-	-	*Priestia* sp.	Pickled radish with thyme from Sątyrz	PX720958
B84/23	-	-	*Priestia* sp.	Pickled radish with thyme from Sątyrz	PX720959
B85/23	-	-	*Priestia* sp.	Pickled radish with thyme from Sątyrz	PX720960
B86/23	-	-	*Lactiplantibacillus* sp.	Pickled radish from Sznajder	PX720961
B87/23	-	-	*Priestia* sp.	Homemade cucumber ferment	PX720962
B88/23	-	-	*Lysinibacillus* sp.	Homemade pickled beets	PX720963
B89/23	-	-	*Bacillus* sp.	Cow’s powdered milk from Siedlce	PX720964
B90/23	-	-	*Bacillus* sp.	Cow’s powdered milk from SM Gostyń	PX720965
B91/23	-	-	*Bacillus* sp.	Cow’s powdered milk from Siedlce	PX720966
B92/23	-	-	*Bacillus* sp.	Cow’s powdered milk from Siedlce	PX720967
B93/23	-	-	*Priestia* sp.	Pickled radish with thyme from Sątyrz	PX720968
B94/23	-	-	*Priestia* sp.	Pickled radish with thyme from Sątyrz	PX720969
B95/23	-	-	*Priestia* sp.	Pickled radish with thyme from Sątyrz	PX720970
B96/23	-	-	*Priestia* sp.	Pickled radish with thyme from Sątyrz	PX720971
B97/23	-	-	*Bacillus* sp.	Cow’s powdered milk from SM Gostyń	PX720972
B98/23	-	-	*Bacillus* sp.	Cow’s powdered milk from Siedlce	PX720973
B99/23	-	+	*Bacillus subtilis*	Homemade pickled beets	PX720974
B100/23	-	-	*Lysinibacillus* sp.	Pickled radish with thyme from Sątyrz	PX720975
B101/23	-	-	*Bacillus* sp.	Cow’s powdered milk from SM Gostyń	PX720976
B102/23	-	-	*Priestia megaterium*	Pickled beets from Lokalny Warzywniak	PX720977
B103/23	-	-	*Bacillus* sp.	Cow’s powdered milk from Mlekovita	PX720978
B104/23	-	-	*Bacillus* sp.	Cow’s powdered milk from Mlekovita	PX720979
B105/23	-	+	*Bacillus subtilis*	Cow’s powdered milk from Mlekovita	PX720980
B106/23	-	+	*Bacillus* sp.	Cow’s powdered milk from Mlekovita	PX720981
B107/23	-	-	*Priestia megaterium*	Pickled beets from Lokalny Warzywniak	PX720982
B108/23	-	-	*Bacillus* sp.	Cow’s powdered milk from Siedlce	PX720983
B109/23	-	-	*Bacillus* sp.	Homemade cucumber ferment	PX720984
B112/23	-	-	*Bacillus* sp.	Cow’s powdered milk from Siedlce	PX720987
B113/23	-	-	*Bacillus* sp.	Cow’s powdered milk from Siedlce	PX720988
B114/23	-	-	*Bacillus* sp.	Powdered unmineralized acid whey from Krasnystaw	PX720989
B115/23	-	-	*Heyndrickxia* sp.	Powdered unmineralized acid whey from Krasnystaw	PX720990
B116/23	-	-	*Bacillus* sp.	Powdered unmineralized acid whey from Krasnystaw	PX720991
B117/23	-	-	*Bacillus* sp.	Powdered unmineralized acid whey from Krasnystaw	PX720992
B118/23	-	-	*Bacillus* sp.	Powdered unmineralized acid whey from Krasnystaw	PX720993
B119/23	-	-	*Bacillus* sp.	Powdered unmineralized acid whey from Krasnystaw	PX720994
B120/23	-	-	*Microbacterium* sp.	Microfiltered fresh pasteurized milk (3.2%) from OSM Garwolin	PX720995
B121/23	-	-	*Bacillus* sp.	Powdered unmineralized acid whey from Krasnystaw	PX720996
B122/23	-	-	*Streptococcus* sp.	Goat’s powdered milk from Danmis	PX720997
B123/23	-	-	*Pseudomonas* sp.	Unsweetened condensed UHT milk (7.5%) from Łaciate	PX720998
B124/23	-	-	*Microbacterium* sp.	Microfiltered fresh pasteurized milk (3.2%) from OSM Garwolin	PX720999
B125/23	-	-	*Microbacterium* sp.	Microfiltered fresh pasteurized milk (3.2%) from OSM Garwolin	PX721000
B127/23	-	-	*Kocuria* sp.	Milk retentate from Krasnystaw dairy	PX721001
B133/23	-	-	*Streptococcus* sp.	Milk retentate from Krasnystaw dairy	PX721002
B135/23	-	-	*Streptococcus* sp.	Milk retentate from Krasnystaw dairy	PX721003
B137/23	-	-	*Streptococcus* sp.	Milk retentate from Krasnystaw dairy	PX721004
B139/23	-	-	*Faucicola* sp.	Milk retentate from Krasnystaw dairy	PX721005
B141/23	-	-	*Streptococcus* sp.	Milk retentate from Krasnystaw dairy	PX721006
B147/23	-	-	*Streptococcus* sp.	Milk retentate from Krasnystaw dairy	PX721007
B149/23	-	-	*Streptococcus* sp.	Milk retentate from Krasnystaw dairy	PX721008
B150/23	-	-	*Faucicola* sp.	Milk retentate from Krasnystaw dairy	PX721009
B152/23	-	-	*Microbacterium* sp.	Milk retentate from Krasnystaw dairy	PX721010
B153/23	-	-	*Microbacterium* sp.	Milk retentate from Krasnystaw dairy	PX721011
B160/23	-	-	*Faucicola* sp.	Milk retentate from Krasnystaw dairy	PX721012
B161/23	-	-	*Microbacterium* sp.	Milk retentate from Krasnystaw dairy	PX721013
B168/23	-	-	*Faucicola* sp.	Milk retentate from Krasnystaw dairy	PX721014
B169/23	-	-	*Bacillus* sp.	Milk retentate from Krasnystaw dairy	PX721015
B170/23	-	-	*Bacillus* sp.	Milk retentate from Krasnystaw dairy	PX721016
B171/23	-	-	*Faucicola* sp.	Milk retentate from Krasnystaw dairy	PX721017
B175/23	-	-	*Faucicola* sp.	Milk retentate from Krasnystaw dairy	PX721018
B176/23	-	-	*Bacillus* sp.	Milk retentate from Krasnystaw dairy	PX721019
B180/23	-	-	*Bacillus* sp.	Milk retentate from Krasnystaw dairy	PX721020
B183/23	-	-	*Faucicola* sp.	Milk retentate from Krasnystaw dairy	PX721021
B185/23	-	-	*Streptococcus* sp.	Milk retentate from Krasnystaw dairy	PX721022
B186/23	-	-	*Faucicola* sp.	Milk retentate from Krasnystaw dairy	PX721023
B189/23	-	-	*Bacillus* sp.	Milk retentate from Krasnystaw dairy	PX721024
B192/23	-	-	*Microbacterium* sp.	Milk retentate from Krasnystaw dairy	PX721025

**Table A2:** Abbreviations and full names of carbon sources and chemical stressors used in the BIOLOG GEN III heatmaps.

Abbreviation	Full Name	Type
A1	Substrate	Carbon source
A2	Dextrin	Carbon source
A3	D-Maltose	Carbon source
A4	D-Trehalose	Carbon source
A5	D-Cellobiose	Carbon source
A6	Gentiobiose	Carbon source
A7	Sucrose	Carbon source
A8	D-Turanose	Carbon source
A9	Stachyose	Carbon source
A10	D-Raffinose	Carbon source
A11	α-D-Lactose	Carbon source
A12	D-Melibiose	Carbon source
A13	β-methyl-D-glucoside	Carbon source
A14	D-Salicin	Carbon source
A15	N-Acetyl-D-Glucosamine	Carbon source
A16	N-Acetyl-β-D-Mannosamine	Carbon source
A17	N-Acetyl-D-Galactosamine	Carbon source
A18	N-Acetyl Neuraminic Acid	Carbon source
A19	α-D-Glucose	Carbon source
A20	D-Mannose	Carbon source
A21	D-Fructose	Carbon source
A22	D-Galactose	Carbon source
A23	3-Methyl Glucose	Carbon source
A24	D-Fucose	Carbon source
A25	L-Fucose	Carbon source
A26	L-Rhamnose	Carbon source
A27	Inosine	Carbon source
A28	D-Sorbitol	Carbon source
A29	D-Mannitol	Carbon source
A30	D-Arabitol	Carbon source
A31	myo-Inositol	Carbon source
A32	Glycerol	Carbon source
A33	D-Glucose-6-PO4	Carbon source
A34	D-Fructose-6-PO4	Carbon source
A35	D-Aspartic Acid	Carbon source
A36	D-Serine	Carbon source
A37	Gelatin	Carbon source
A38	Glycyl-L-Proline	Carbon source
A39	L-Alanine	Carbon source
A40	L-Arginine	Carbon source
A41	L-Aspartic Acid	Carbon source
A42	L-Glutamic Acid	Carbon source
A43	L-Histidine	Carbon source
A44	L-Pyroglutamic Acid	Carbon source
A45	L-Serine	Carbon source
A46	Pectin	Carbon source
A47	D-Galacturonic Acid	Carbon source
A48	L-Galactonic Acid Lactone	Carbon source
A49	D-Gluconic Acid	Carbon source
A50	D-Glucuronic Acid	Carbon source
A51	Glucuronamide	Carbon source
A52	Mucic Acid	Carbon source
A53	Quinic Acid	Carbon source
A54	D-Saccharic Acid	Carbon source
A55	p-Hydroxy Phenylacetic Acid	Carbon source
A56	Methyl Pyruvate	Carbon source
A57	D-Lactic Acid Methyl Ester	Carbon source
A58	L-Lactic Acid	Carbon source
A59	Citric Acid	Carbon source
A60	α-Keto-Glutaric Acid	Carbon source
A61	D-Malic Acid	Carbon source
A62	L-Malic Acid	Carbon source
A63	Bromo-Succinic Acid	Carbon source
A64	Tween 40	Carbon source
A65	γ-Aminobutyric Acid	Carbon source
A66	α-Hydroxy Butyric Acid	Carbon source
A67	β-Hydroxy-D,LButyric Acid	Carbon source
A68	α-Keto-Butyric Acid	Carbon source
A69	Acetoacetic Acid	Carbon source
A70	Propionic Acid	Carbon source
A71	Acetic Acid	Carbon source
A72	Formic Acid	Carbon source
A73	Mean value	-
A74	Aztreonam	Chemical stressor
A75	Sodium Butyrate	Chemical stressor
A76	Sodium Bromate	Chemical stressor
A77	pH 6	Chemical stressor
A78	pH 5	Chemical stressor
A79	1% NaCl	Chemical stressor
A80	4% NaCl	Chemical stressor
A81	8% NaCl	Chemical stressor
A82	1% Sodium Lactate	Chemical stressor
A83	Fusidic Acid	Chemical stressor
A84	D-serine	Chemical stressor
A85	Troleandomycin	Chemical stressor
A86	Rifamycin SV	Chemical stressor
A87	Minocycline	Chemical stressor
A88	Lincomycin	Chemical stressor
A89	Guanidine HCl	Chemical stressor
A90	Niaproof 4	Chemical stressor
A91	Vancomycin	Chemical stressor
A92	Tetrazolium Violet	Chemical stressor
A93	Tetrazolium Blue	Chemical stressor
A94	Nalidixic Acid	Chemical stressor
A95	Lithium Chloride	Chemical stressor
A96	Potassium tellurite	Chemical stressor
A97	Positive control	Chemical stressor

**Table A3:** Morphological characteristics of the examined bacterial strains determined by holotomography imaging.

Strain	Taxon	Cell Morphology	Approximate Cell Size (µm)	Morphological Characteristics
B56/23	*B. subtilis*	Moderately elongated rod-shaped cells	~3–4 × 0.7–0.9	Morphology typical of the *B. subtilis* group; variable chain formation and occasional clustering were observed within this group
B64/23	*B. subtilis*	Elongated rod-shaped cells	~4 × 0.7–0.9	More elongated and slightly larger than B56/23
B80/23	*B. subtilis*	Smaller rod-shaped cells with reduced elongation	~3 × 0.5–0.9	Smaller cells compared with the other examined *B. subtilis* strains
B99/23	*B. subtilis*	Rod-shaped cells	~3.5 × 0.7–0.9	Morphology consistent with the examined *B. subtilis* group
B105/23	*B. subtilis*	Rod-shaped cells	~4 × 0.7–0.9	Morphology consistent with the examined *B. subtilis* group
B107/23	*P. megaterium*	Large rod-shaped cells	~6 × 1–1.2	Short chains and multilayered structures; heterogeneous intracellular density; markedly larger than the remaining isolates
